# Syndecan-4/PAR-3 signaling regulates focal adhesion dynamics in mesenchymal cells

**DOI:** 10.1186/s12964-020-00629-3

**Published:** 2020-08-18

**Authors:** Alejandra Valdivia, Areli Cárdenas, Marianne Brenet, Horacio Maldonado, Milene Kong, Jorge Díaz, Keith Burridge, Pascal Schneider, Alejandra San Martín, Rafael García-Mata, Andrew F. G. Quest, Lisette Leyton

**Affiliations:** 1 Cellular Communication Laboratory, Program of Cellular & Molecular Biology, Instituto de Ciencias Biomédicas, Facultad de Medicina, Universidad de Chile, Av. Independencia 1027, Independencia, 838-0453 Santiago, Chile; 2 Center for studies on Exercise, Metabolism and Cancer (CEMC) and Advanced Center for Chronic Diseases (ACCDiS), Facultad de Medicina, Universidad de Chile, 838-0453 Santiago, Chile; 3 Microscopy in Medicine (MiM) Core, Emory University, Atlanta, GA 30322 USA; 4 Department of Cell Biology and Physiology, University of North Carolina at Chapel Hill, Chapel Hill, NC 27599 USA; 5 School of Medicine, Division of Cardiology, Emory University, Atlanta, GA 30322 USA; 6 Department of Pediatrics, Pulmonology Division, Program for Rare and Interstitial Lung Disease, University of North Carolina at Chapel Hill, Chapel Hill, NC 27599 USA; 7 UNC Catalyst for Rare Disease, UNC Eshelman School of Pharmacy, University of North Carolina at Chapel Hill, Chapel Hill, NC 27599 USA; 8 Departamento Biomédico, Facultad de Ciencias de la Salud, Universidad de Antofagasta, Antofagasta, Chile; 9 Department of Biochemistry, University of Lausanne, 1066 Epalinges, Switzerland; 10 Department of Biological Sciences, University of Toledo, Toledo, OH 43606 USA

**Keywords:** Focal adhesion turnover, Cytoskeleton, Cell polarity, Mesenchymal cell migration, Wound healing

## Abstract

**Background:**

Syndecans regulate cell migration thus having key roles in scarring and wound healing processes. Our previous results have shown that Thy-1/CD90 can engage both αvβ3 integrin and Syndecan-4 expressed on the surface of astrocytes to induce cell migration. Despite a well-described role of Syndecan-4 during cell movement, information is scarce regarding specific Syndecan-4 partners involved in Thy-1/CD90-stimulated cell migration.

**Methods:**

Mass spectrometry (MS) analysis of complexes precipitated with the Syndecan-4 cytoplasmic tail peptide was used to identify potential Syndecan-4-binding partners. The interactions found by MS were validated by immunoprecipitation and proximity ligation assays. The conducted research employed an array of genetic, biochemical and pharmacological approaches, including: PAR-3, Syndecan-4 and Tiam1 silencing, active Rac1 GEFs affinity precipitation, and video microscopy.

**Results:**

We identified PAR-3 as a Syndecan-4-binding protein. Its interaction depended on the carboxy-terminal EFYA sequence present on Syndecan-4. In astrocytes where PAR-3 expression was reduced, Thy-1-induced cell migration and focal adhesion disassembly was impaired. This effect was associated with a sustained Focal Adhesion Kinase activation in the siRNA-PAR-3 treated cells. Our data also show that Thy-1/CD90 activates Tiam1, a PAR-3 effector. Additionally, we found that after Syndecan-4 silencing, Tiam1 activation was decreased and it was no longer recruited to the membrane. Syndecan-4/PAR-3 interaction and the alteration in focal adhesion dynamics were validated in mouse embryonic fibroblast (MEF) cells, thereby identifying this novel Syndecan-4/PAR-3 signaling complex as a general mechanism for mesenchymal cell migration involved in Thy-1/CD90 stimulation.

**Conclusions:**

The newly identified Syndecan-4/PAR-3 signaling complex participates in Thy-1/CD90-induced focal adhesion disassembly in mesenchymal cells. The mechanism involves focal adhesion kinase dephosphorylation and Tiam1 activation downstream of Syndecan-4/PAR-3 signaling complex formation. Additionally, PAR-3 is defined here as a novel adhesome-associated component with an essential role in focal adhesion disassembly during polarized cell migration. These novel findings uncover signaling mechanisms regulating cell migration, thereby opening up new avenues for future research on Syndecan-4/PAR-3 signaling in processes such as wound healing and scarring.

**Graphical abstract:**

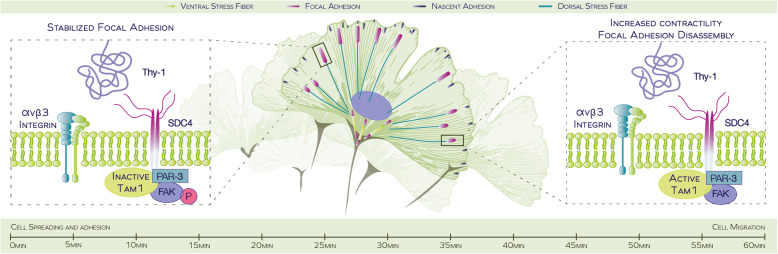

## Plain English summary

Cell movement is crucial during wound healing and scaring processes. To move, cells need to coordinately protrude and form points of adhesion (or focal adhesions) with the surrounding matrix (substrate) at one end of their body (head) and disassemble these focal adhesions on the opposite side (tail). Then, small fibers located in the cell body can pull the tail of the cell towards the head, to move the cell forward, in a repetitive process of protrusion–adhesion–contraction–detachment. Different proteins present on the cell surface regulate cell movement by coordinating adhesion of the cells to the substrate. Our previous studies show that Thy-1/CD90, a protein found in neurons, activated endothelium and some fibroblasts, regulates cell movement through its interaction with two other membrane proteins: integrin and Syndecan-4. Here, we describe the mechanism by which Thy-1/CD90 controls such movement, by unveiling a novel interaction between Syndecan-4 and PAR-3, a protein that has been involved in cell polarity. When this interaction was blocked, for example after decreasing the amount of PAR-3 or Syndecan-4, we found that cells did not move, because they were not capable of disassembling those points of contact with the substrate, thus impeding the cyclic process of cell motility.

## Background

Focal adhesion (FA), a key type of adhesive cell and extracellular matrix contact, is relevant for both mechanical and biochemical signaling [1]. The binding of transmembrane proteins (e.g., integrins and Syndecan-4) to extracellular ligands and the cytoskeleton mediates FA formation, thus promoting cell adhesion. A critical component in the cell-adhesion process is the adhesome –i.e., a complex of > 180 proteins and associated effector partners that connect with receptors such as integrins and Syndecan-4 [2, 3] regulating not only cell adhesion but also other cellular processes, including cell polarization, and migration. Cell migration is a cyclical process that first requires cell polarization and the formation of membrane protrusions at the leading edge. Then, FA and actin microfilament bundles (i.e., stress fibers) form, and cells contract/retract the rear end to promote forward movement [4, 5]. Many receptors and proteins are involved in cell movement, nevertheless; the key signaling components of the adhesome that regulate FA dynamics remain poorly defined.

An integrin and Syndecan-4 cellular counterpart possessing an integrin-binding site and a heparin-binding domain (HBD) is the neuronal surface protein Thy-1/CD90, a protein included in the integrin adhesome by virtue of the Thy-1/β3 integrin interaction described initially by our group [6, 7]. Interestingly, Thy-1/CD90 HBD has also been involved in the formation of FA in astrocytes through the engagement of the proteoglycan Syndecan-4 in a cooperative interaction with αvβ3 integrin [8].

Syndecan-4 possesses a cytoplasmic domain with two conserved regions flanking a central variable region that binds to phosphatidylinositol 4,5-bisphosphate (PIP_2_) [9–11] and PKCα [12–14] and this complex has been implicated in FA formation [15, 16]. In fibroblasts, available evidence indicates that the small GTPase Rac1 localizes to the cell leading edge after stimulation with Fibronectin in a PKCα-dependent manner, indicating that Rac1 is the main GTPase activated downstream of Syndecan-4 ligation [13]. In our model of Thy-1/CD90-induced astrocyte migration, Syndecan-4 engagement by Thy-1/CD90 activates FAK/Rac1, which are required to induce astrocyte polarization and migration over the extracellular matrix (ECM) [8, 17]. However, the signaling partners of Syndecan-4 linking this proteoglycan to the FAK/Rac1 pathway are still unknown.

The cytoplasmic C2 domain of Syndecan-4 possesses an EFYA C-terminal sequence, which binds proteins containing the post synaptic density protein, *Drosophila* disc large tumor suppressor, and zonula occludens-1 protein (PDZ) domains such as Syntenin, CASK, synectin, synbindin and the Rac1 guanine nucleotide exchange factor (GEF) Tiam1 [18, 19]. To learn more about other possible partners of Syndecan-4, here we performed mass spectrometry of complexes precipitated with Syndecan-4 cytoplasmic tail peptides and found the adaptor protein PAR-3 as a new binding partner for Syndecan-4.

Partitioning-defective (PAR) proteins play roles in the polarized cell migration of astrocytes, fibroblasts, and T-cells [20, 21, 27]. For example, the leading edge of motile cells present the PAR-6/aPKC polarization complex, which is needed to reorient the microtubule-organizing center, the microtubule cytoskeleton, and membrane trafficking towards the leading edge [22–24]. Particularly notable among the PAR proteins is PAR-3, a protein related to cell polarity in several cell types [23, 25, 26]. Most interestingly, PAR-3 possesses three PDZ domains and binds to Rac1 GEFs at the leading edge of cells, thereby spatiotemporally controlling Rac1 activity and cell migration [26, 27]. While polarized cell migration clearly depends on PAR proteins, a possible role for PAR-3 as a downstream effector of Syndecan-4 has yet to be determined.

In adult individuals, mesenchymal cells are relatively quiescent cells that help maintain connective tissue and regulate homeostatic functions, such as stabilizing even the smallest blood vessels [28]. Tissue damage or inflammatory processes activate these cells, with fibroblasts transforming into myofibroblasts and astrocytes into reactive astrocytes [29]. These transformed cells are motile and scar-forming. Although an initially beneficial process, scarring requires fine-tuned regulations for appropriate tissue repair [30–33]. Thus, understanding and influencing the wound-healing process necessarily requires complete knowledge on the molecular mechanisms governing cell migration.

Here, we used astrocytes and fibroblasts to study the involvement of Syndecan-4 and PAR-3 in cell adhesion/migration. The obtained research data identify a novel signaling pathway, termed Syndecan-4/PAR-3, involved in controlling Thy-1/CD90-induced FA dynamics in mesenchymal cells by regulating Focal Adhesion Kinase (FAK) autophosphorylation and Tiam1 activation. PAR-3 binding to Syndecan-4 further suggest that PAR-3 is a novel adhesome-associated component with an essential role in polarized cell migration.

## Methods

### Cell culture

The rat astrocytic cell line DI TNC1 (ATCC CRL-2005) was maintained in RPMI-1640 medium (GIBCO Life Technologies) as previously described [17]. CAD cells expressing or not Thy-1/CD90 were grown in Dulbecco’s modified Eagle’s medium: Nutrient Mixture F-12 medium (DMEM/F12) (Gibco) containing 8% FBS and 1% penicillin-streptomycin. Cells with silenced Thy-1/CD90 where maintained in the same medium containing 0.5 μg/ml puromycin (Sigma-Aldrich) [7, 34]. As reported [35] MEFs were prepared from E13.5 C57BL/6 mouse embryos. Primary MEFs (passage 2) were immortalized by transfecting them with SV40 large T-antigen (Addgene plasmid #13970) using Fugene HD (Promega). Cells were seeded at a ratio of 1:10 for 9 additional passages upon reaching confluence. Immortalized MEFs were grown in DMEM-high glucose supplemented with 10% FBS (Atlanta Biological), 1% penicillin-streptomycin and 1% Glutamax (Gibco) and used for experiments until passage 17.

### Plasmids and transfections

Retroviral expression vector (pBabe-puro) encoding Syndecan-4 fused to an extracellular HA-tag was kindly provided by Dr. Mark Morgan (University of Liverpool, UK) [36]. Syndecan-4-HA fragment was subcloned into EcoRI/XhoI sites of pcDNA3.1(+) plasmid. For the live cell experiments we used mCherry-vinculin (modified pEGFP-C1-mCherry plasmid harboring a deletion of the GFP gene sequence), kindly provided by Dr. Vicente Torres (Universidad de Chile, Chile) [37]. pEGFP-C1-Tiam1-PHnCCEx containing a mutation in the RhoK phosphorylation site encoding for a dominant negative mutant of Tiam1 was kindly provided by Dr. María Paz Marzolo (P. Universidad Católica de Chile, Chile) [38].

To downregulate PAR-3 expression we used siRNA ON-TARGET plus SMART pool (Thermo Scientific) against rat PAR-3 or FlexiTube siRNA Mm_Pard3_4 (Qiagen) targeting mouse PAR-3. A mix of two Tiam1 siRNA targeting the human Tiam1 with 88% homology with the rat Tiam1 sequence (Dharmacon) was used to lower Tiam1 expression. *Silencer*® Negative Control No. 1 (Ambion™) or AllStars (Qiagen) were used as siRNA negative control. Plasmids and siRNAs transfections of DI TNC1 cells were performed with Amaxa Nucleofector following manufacturer’s recommendations for astrocytes (Amaxa Biosystems, Lonza). Lipofectamine RNAiMAX Reagent and Lipofectamine 3000 (Thermo Fisher Scientific) were used to transfect MEFs.

### Antibodies and reagents

The recombinant protein Thy-1-Fc and TRAIL-R2-Fc were obtained as reported [6, 8, 39], and used coupled to protein A (Sigma-Aldrich) for cell stimulation. Complexes were incubated in a 10:1 (recombinant fusion protein: Protein-A) ratio for 60 min at 4 °C, shaking gently [40]. Immunofluorescence reagents were rhodamine-conjugated phalloidin and DAPI (diamidino-2-phenylindole) (Sigma-Aldrich). Antibodies were rabbit anti-PAR-3 (Merck, Millipore, and ProteinTech), mouse anti-HA mAb and rabbit anti-giantin (Covance Research Products), mouse anti-vinculin mAb and anti β-actin from Sigma-Aldrich, and goat anti-mouse IgG conjugated to Alexa fluor 488, goat anti-mouse IgG or goat anti-rabbit IgG conjugated to Alexa fluor 546 (Molecular Probes). Other antibodies used were rabbit anti-Syntenin (Synaptic Systems), rabbit anti-Syndecan-4 (Abbexa) and rabbit anti-Tiam1 (Santa Cruz Biotechnology), rabbit anti-p-FAK (Tyr397) (Upstate Biotechnology), rabbit anti-FAK (Cell Signaling), and horseradish peroxidase-conjugated goat anti-mouse IgG polyclonal antibody (Bio-Rad Laboratories, Inc.) or goat anti-rabbit IgG, mouse anti-rabbit IgG light chain specific and goat anti-rabbit IgG Fc specific (Jackson ImmunoResearch Labs, Inc).

### Immunoprecipitation and Western blotting

DI TNC1 cells were serum-deprived for 16 h and stimulated or not for 30 min with a complex of Thy-1-Fc or TRAIL-R2-Fc conjugated to Protein A, or 3% FBS as a migration-inducing control. Then, cells were lysed (50 mM Hepes-KOH, pH 7.4, 50 mM NaCl, 750 μM CaCl_2_, 1 mM MgCl_2_, 1% Triton X-100, 2 mM DTT) supplemented with protease and phosphatase inhibitor cocktail (Biotool) and pre-incubated with streptavidin-coated Sepharose beads to clean lysates of biotin containing proteins. Soluble fraction of lysates was incubated with streptavidin-Sepharose beads coupled to biotinylated peptides (15 μg/sample) corresponding to 10 amino acids of the cytoplasmic tail of Syndecan-4 (K K A P T N E F Y A) or control peptides in which the last 4 amino acids were replaced by glycine (K K A P T N G G G G) [41]. Samples were separated by SDS-PAGE and silver stained. In-gel bands corresponding to Syndecan-4 tail-precipitated proteins that were absent in the control condition (modified peptides) were extracted from the gel, digested and analyzed by liquid chromatography-tandem mass spectrometry (UNC Michael Hooker Proteomics Center-UNC, Chapel Hill). Data, obtained from two independent experiments, were searched against publicly available databases using Mascot search engine. In a different set of experiments, precipitated proteins were immunoblotted for PAR-3 and Syntenin to corroborate mass spectrometry results.

For Syndecan-4 immunoprecipitation, cells were transfected with Syndecan-4-HA plasmid; 48 h later, cells were serum-starved for 16 h and stimulated for 30 min or not with Thy-1-Fc or TRAIL-R2-Fc conjugated to Protein A. Cells were lysed (20 mM Tris-HCl, pH 7.4, 0.15 N NaCl and 0.5% NP-40, supplemented with protease and phosphatase inhibitor cocktail) and incubated with a mouse anti-HA antibody. Precipitated proteins were analyzed by Western blotting for PAR-3, FAK, Syndecan-4 and Syntenin using rabbit polyclonal antibodies. Alternatively, MEFs were transfected with Syndecan-4-HA plasmid and selected with G418 (1.65 mg/ml). Cells were plated at sub-confluency, serum-starved for 16 h and stimulated with Thy-1-Fc-Protein A beads for 30 min or 3% FBS for 3 min. Cell lysates were incubated with rabbit anti-PAR-3 antibody and precipitated proteins were analyzed by Western blotting for HA (Syndecan-HA, mouse anti HA antibody) and Syntenin (rabbit antibody). An HRP-conjugated goat anti-rabbit IgG, Fc specific secondary antibody was used to detect the anti-Syntenin antibody and avoid interferences by the light chain immunoglobulin used to precipitate PAR-3. Antibody detection was performed using an enhanced ECL detection system (GE Healthcare). The intensity of the bands was quantified by using ImageJ (National Institutes of Health).

### Proximity ligation assay (PLA)

DI TNC1 cells were transfected with Syndecan-4-HA or control plasmids. After 48 h, cells were serum-starved for 30 min and stimulated or not with Thy-1-Fc or TRAIL-R2-Fc conjugated to Protein A for 30 min. Cells were fixed and permeabilized with 0.1% Triton for 10 min. PLA was performed using DuoLink PLA probes and reagents (Sigma-Millipore) following the manufacturers protocol. Signal puncta was analyzed using the Analyze Particles function in ImageJ software as described below.

### Indirect immunofluorescence

Cells were fixed at the indicated time points, rinsed, permeabilized and blocked as previously reported [8]. Cells were stained with appropriate antibody as described in each figure legend and coverslips were washed and mounted using 10% Mowiol-2.5% 1,4-Diazabicyclo [2.2.2] octane. Samples were visualized using a confocal Nikon Spectral C2 Plus microscope (Plan-Apo NA1.4 60x oil) or a Zeiss LSM 510 META Laser Scanning Confocal Microscope (Plan-Apo 420,782–9900 63x oil).

### PAR-3 and Syndecan-4 immunofluorescence

DI TNC1 cells were transfected with Syndecan-4-HA and 15.000 cells were seeded on coverslips in 24-well plates for 24 h. Then, cells were starved for 30 min and stimulated or not with a complex Thy-1-Fc or TRAIL-R2-Fc (4 μg/well) [6, 39] conjugated to Protein A in a 10:1 ratio, for 60 min. Next, cells were processed by immunofluorescence as described [42].

### Wound healing and polarity assays

DI TNC1 cells were transfected and seeded in 24-well plates for 24 h at 70–80% confluence. For wound healing assays, cells were transfected with siRNA targeting PAR-3, Tiam1 or control, or with DN-Tiam1 (dominant negative for Tiam1), and for polarity assays, with siRNA targeting PAR-3 or siRNA control. DI TNC1 monolayers were scratched with a sterile micropipette tip; then, attached cells were washed twice with PBS and left in serum-free RPMI media for 30 min before the addition of the recombinant proteins to stimulate migration. Cells were stimulated for 7 (polarity) or 24 h (wound-healing) with Thy-1-Fc or TRAIL-R2-Fc conjugated to Protein A as described [43]. Since cells are kept in serum-free media during the stimulation period, no proliferation effect is foreseen, as we have previously shown [17]. The cell-free area was measured at time zero (before applying the stimulus) and at 24 h by phase contrast microscopy to quantify wound closure [43] using ImageJ software [44]. In cell polarization experiments, samples were fixed after stimulation and stained for nucleus (DAPI) and the Cis-medial Golgi protein giantin (rabbit anti-giantin from Covance Research Products). Cells were considered polarized when the Golgi signal was localized in front of the nucleus within an angle of 120° (see Fig. 2b). One hundred cells were monitored per condition, and cell polarization was evaluated as the percentage of cells along the wound border exhibiting polarized Golgi structures [45].

### Far-indirect immunofluorescence for active GEFs

DI TNC1 cells were transfected with siRNA PAR-3 or siRNA control and seeded in 8-well Falcon culture slides (Corning) for 24 h at 50% confluence. Cells were serum-starved for 30 min and stimulated or not with Thy-1-Fc or TRAIL-R2-Fc conjugated to Protein A. Then, cells were subjected to a modified protocol of the original Far-immunofluorescence method previously described [46]. Briefly, cells were fixed, permeabilized and blocked as for regular immunofluorescence [43]. Samples were then incubated with GST-Rac1G15A or GST purified recombinant protein for 60 min and washed. Subsequently, samples were incubated with anti-GST antibody followed by secondary Alexa Fluor 546 goat anti-rabbit. GST-recombinant proteins were prepared according to the original report [47]. Immunofluorescence obtained with GST only was considered as background and subtracted from all samples. Similarly, in other experiments cells were stained for GST and Tiam to determine the levels of Tiam activation in situ in astrocytes. The co-localization between GST-Rac1G15A with Tiam was analyzed using ImageJ [44].

### Focal adhesion analysis

DI TNC1 cells were transfected with siRNA PAR-3 or siRNA control. For video microscopy siRNAs were co-transfected with mCherry-vinculin. Cells were then seeded in coverslips in 24-well plates for assembly and disassembly assays, or in 8-well Lab-Tek II chambered coverglass (Nunc) for time-lapse video microscopy. For assembly assays, cells were serum-starved for 30 min and stimulated or not with Thy-1-Fc-Protein A or TRAIL-R2-Fc-Protein A for 15 min. For disassembly assays, cells were serum-starved during 30 min and treated with 10 μm Nocodazole (Sigma, Aldrich) for 4 h (to disrupt microtubules); then, Nocodazole was removed (time zero [t0]) promoting polymerization of microtubules and disassembly of FAs. The stimuli (Thy-1-Fc-Protein A or its control TRAIL-R2-Fc-Protein A) were applied for different time points (0–60 min) as indicated. Cells were fixed and immunofluorescence with anti-vinculin antibody was performed. For time-lapse video microscopy images were captured starting at time zero up to 30 min (every 1 min) in a temperature controlled chamber assembled in an epifluorescence Nikon Eclipse *Ti* microscope. Kinetics of focal adhesions was measured from these time-lapse experiments, scoring at least 10 mCherry-positive focal adhesions per experiment.

Similarly, experiments to follow FA dynamics in MEFs were performed as described above, but cells were co-transfected with siRNA targeting PAR-3 or siRNA control and siGlo RNAi (GE Dharmacon). Cells were then treated with 0,1 μm Nocodazole for 4 h and stimulated with Thy-1-Fc-Protein A or TRAIL-R2-Fc-Protein A as described for astrocytes.

Quantification of focal adhesion area and number was performed following previously described protocols [42]. The average number of FAs as well as the average area per FA was determined for at least 50 cells in each experimental condition using the ImageJ/Fiji program and the function ‘Analyze particles’ [44].

### FAK autophosphorylation

Cells were transfected or not with siRNA PAR-3 or siRNA control and seeded in 24-well plates for 24 h at 50% confluence for DI TNC1. Cells were manipulated as described for FA disassembly assay (see above). The stimuli (Thy-1-Fc-Protein A or TRAIL-R2-Fc-Protein A) were applied for different time points (0–60 min). Then, cells were lysed in Laemmli buffer containing the protease and phosphatase inhibitor cocktail, analyzed by Western blot (see above) for p-FAK (Tyr397) and total FAK.

### Tiam1 activity

DI TNC1 cells were grown in 10 cm plates, and Tiam1 activity was measured using an affinity precipitation assay previously described [48]. Briefly, cells were serum-starved for 16 h and subsequently stimulated with previously fixed CAD (shThy-1) or CAD (shLuc) cells as reported [39] for different periods of time. Thy-1/CD90 is highly expressed on the surface of CAD cells and this mode of activation, previously validated by our group, is used when a large amount of Thy-1-Fc protein is required to stimulate cells [17]. After stimulation, cells were washed and scraped in lysis buffer (25 mM HEPES, pH 7.4, 0.1 M NaCl, 1% NP40, 5 mM MgCl_2_, 10% glycerol, 1 mM DTT, 10 μg/ml leupeptin, 10 μg/ml aprotinin and 1 mM sodium orthovanadate). Next, cell lysates were incubated with 30 μg of GST-Rac1G15A bound to GSH-agarose beads for 1 h at 4 °C and mixed gently on a rocking shaker. After washing, beads were resuspended in Laemmli buffer. Samples were resolved by SDS-PAGE, transferred to nitrocellulose membrane, which was blocked with 5% fat-free milk in 0.1% Tween-PBS, and incubated with anti-Tiam1 (1:1000). Active Tiam1 was determined by Western blot analysis from the precipitated fraction and normalized to total protein (input).

### Statistical analysis

All data are expressed as the mean ± standard error of mean (mean ± s.e.m.) from three independent experiments (*n* = 3). For each experimental condition at least 50 cells were evaluated per every independent experiment. Data were compared through non-parametric Mann-Whitney analysis, 1-way or 2-way ANOVA, accordingly. Significant differences (*) were established at *P* < 0.05**.**

## Results

### PAR-3 interacts with Syndecan-4

The C-terminal PDZ-binding domain of Syndecan-4 (C2) associates with various PDZ-containing proteins or other molecules involved in cell migration [18]. Therefore, polarized cell migration was stimulated with fetal bovine serum (FBS) to identify C2-recruited Syndecan-4 binding proteins. The astrocyte cell line DI TNC1 was selected as a mesenchymal-type cell model based on prior use in studies related to cell adhesion and migration [6–8, 17, 43]. DI TNC1 cells were first stimulated with FBS, and lysates precipitated using streptavidin-Sepharose beads coupled to biotinylated Syndecan-4 peptides corresponding to the C-terminal last 10 amino acids of the cytoplasmic domain (Additional file 1A). As a control peptide, the same 10 amino acids with the last 4 amino acids (EFYA) replaced by glycine were used [41]. The associated proteins were separated in an SDS-PAGE gel and Silver stained (Additional file 1B). The bands of interest were cut from the gel and identified by mass spectrometry. Astrocytes stimulated with FBS presented various proteins binding to the Syndecan-4 cytoplasmic peptide. PAR-3 was one of the detected Syndecan-4 binding proteins, making this the first study to describe such an interaction (Table 1). Other proteins included Syntenin (AF248548) (Table 1), a protein that serves as a good control due to its known Syndecan-4 C2 domain binding properties [49]. Syntenin directly interacts with the Syndecan-4 EFYA sequence, making it a perfect experimental control for the specificity of the biotinylated peptides used in the precipitation assays [18]. Noteworthy, because these experiments are performed adding excess of ectopic peptides (15 μg/sample), no competition between PAR-3 and Syntenin for the biotinylated peptides is foreseen.

**Table 1:** Mass spectrometry data showing the identity of the proteins precipitated with biotinylated peptides of the Syndecan-4 cytoplasmic tail

Protein Name	Species	Database Accession ID	MW (Da)	Peptide Count^a^	MS & MS/MS Score^b^	Peptide Sequenced Ion Score^c^	Scoring Threshold^d^
Atypical protein kinase C isotype-specific interacting (PAR-3)	*Rattus norvegicus*	Q9Z340.1	149,356.5	36	315	177	56
Tubulin, alpha 1C	*Rattus norvegicus*	AAH78829.1	49,905.5	8	185	159	56
Tubulin beta chain 15	*Rattus norvegicus*	Q3KRE8.1	49,904.9	9	151	115	56
Insulin-like growth factor binding protein 5 protease	*Rattus norvegicus*	AAD52683.1	51,297.7	12	227	191	56
Type I keratin KA10	*Rattus norvegicus*	DAA04466.1	56,470.5	7	69	53	56
Actin beta	*Rattus norvegicus*	AAH63166.1	41,723.7	20	635	487	56
Heterogeneous nuclear ribonucleoprotein A2/B1/B0	*Mus musculus*	AAN16352.1	37,379.7	11	101	58	78
Syntenin-1	*Rattus norvegicus*	NP_114192.1	32,402.7	14	384	299	56
Streptavidin core, residues 13,139 pH 4.5, chain B	*Streptomyces avidinii*	1SWAB2	9191.5	6	549	498	78

^**a**^Number of peptides that match the theoretical digest of the primary protein identified

^**b**^Quality score of peptide-mass fingerprint matches and of MS/MS peptide fragment ion matches, if MS/MS data were generated

^**c**^Quality score of only MS/MS peptide fragment ion matched, if MS/MS data were generated

^**d**^Significant score threshold. For each given database, a hit with an “MS & MS/MS Score” above the indicated value was considered a significant identification (*p* < 0.05). Only hits with an “MS & MS/MS Score” above this value are reported

**Fig. 1:**
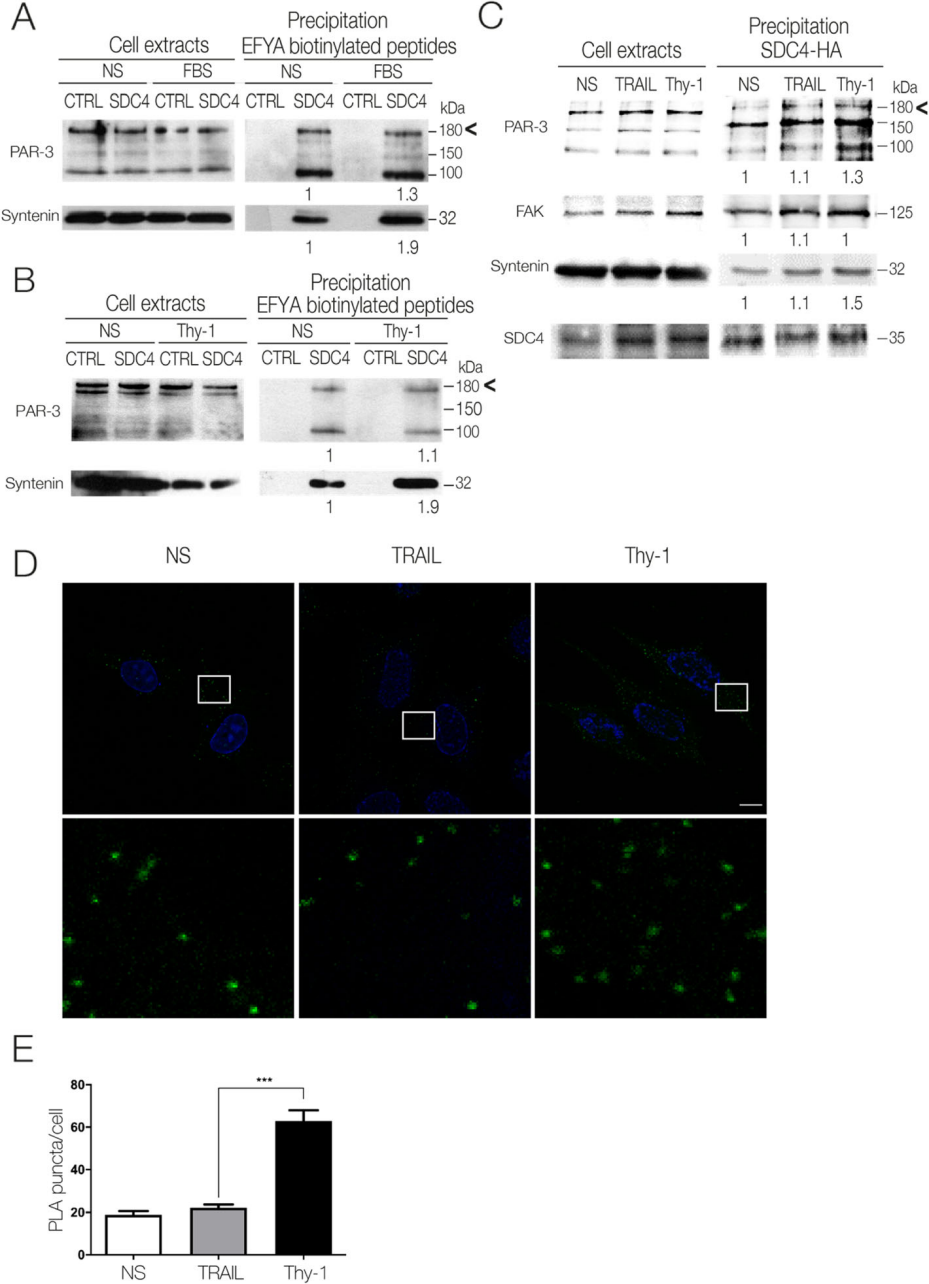
Thy-1 treatment induced association of PAR-3 with Syndecan-4 in DI TNC1 astrocytes. **a** Cell lysates obtained upon FBS or (**b**) Thy-1-Fc-Protein A (Thy-1) stimulation were precipitated using biotinylated peptides consisting in the 10 amino acids of Syndecan-4 cytoplasmic domain (SDC4) or control peptides, in which the last 4 amino acids (EFYA) were replaced by glycine. Samples were immunoblotted for PAR-3. Syntenin was used as a precipitation control since it interacts directly with the Syndecan-4 EFYA sequence. **c** Syndecan-4-HA transfected DI TNC1 cells were stimulated with Thy-1-Fc-Protein A (Thy-1) or TRAIL-R2-Fc-Protein A (TRAIL) as a negative control. Cell lysates were immunoprecipitated with the anti-HA antibody and immunoblotted for PAR-3, FAK, Syndecan-4, or Syntenin. **d** Association between PAR-3 and Syndecan-4 was evaluated by Proximity Ligation Assay (PLA). DI TNC1 cells were transfected with Syndecan-4-HA or with the empty plasmid. Subsequently, cells were serum starved for 30 min and stimulated or not (NS) with Thy-1-Fc-Protein A (Thy-1) or TRAIL-R2-Fc-Protein A (TRAIL), fixed and processed for PLA using antibodies against PAR-3 or HA. Pictures are representative of PLA puncta observed for each condition as indicative of the interaction between PAR-3 and Syndecan-4-HA. Lower panels show magnification of the indicated area. Magnification bar = 10 μM. **e** Graph shows the number of PLA puncta per cell. ***P < 0.001

To confirm PAR-3/Syndecan-4 binding, the obtained lysate precipitates were also analyzed through Western blotting with the anti-PAR-3 and anti-Syntenin antibodies. Full length PAR-3 (180 kDa) and Syntenin were detected when precipitating with wild-type but not control Syndecan-4 peptides in non-stimulated cells (Fig. 1a and b). Cell stimulation with FBS or Thy-1-Fc (a fusion protein of the cell adhesion molecule Thy-1/CD90) increased Syntenin precipitation with the biotinylated wild-type peptide, but PAR-3 precipitation was unaffected or only slightly affected (Fig. 1a and b). Similarly, when astrocytes transfected with hemagglutinin (HA)-tagged Syndecan-4 were used for Syndecan-4 immunoprecipitation, we observed a modest increase in full length PAR-3 co-precipitated in Thy-1-Fc-stimulated cells than in non-stimulated or cells treated with TRAIL-R2-Fc (Fig. 1c). As in previous studies [6, 43], we used a non-related protein (TRAIL-R2) fused to a Fc fragment (TRAIL-R2-Fc) as a negative control of the fusion protein Thy-1-Fc. We have previously shown that TRAIL-R2-Fc neither binds to cell surface receptors nor elicits cellular responses [8, 17, 43, 50]. Additionally, as part of the complex precipitating with Syndecan-4-HA, we found Syntenin and FAK, two proteins involved in cell migration and known to interact with PAR-3 [51] (Fig. 1c). Syndecan-4 was also detected as an immunoprecipitation control in these experiments. Furthermore, in situ Proximity Ligation Assay (PLA) showed that PAR-3 interacts very closely (< 40 nm) with Syndecan-4 in Syndecan-4-HA-transfected astrocytes (Fig. 1d). The abundance of this interaction was also significantly increased after Thy-1-Fc stimulation, but not in non-stimulated cells or cells treated with TRAIL-R2-Fc (Fig. 1e).

Altogether, these results indicate that PAR-3 binds to Syndecan-4 and that its interaction could be regulated to a certain extend during FBS- or Thy-1/CD90-induced cell migration.

### PAR-3 is involved in Thy-1/CD90-induced cell adhesion and migration, but not polarization

Prior research reports indicate that neuronal Thy-1/CD90 engages Syndecan-4 in a cooperative interaction with αvβ3 integrin to initially induce FA formation in astrocytes (~ 30 min). Sustained Thy-1/CD90 stimulation leads to Rac1 activation and astrocyte migration (> 60 min) [8, 17]*.* Considering that PAR-3 and Syndecan-4 form a complex in astrocytes, termed herein as the Syndecan-4/PAR-3 signaling complex, and that the abundance of such complexes changes in Thy-1/CD90-stimulated cells (Fig. 1d and e), the participation of PAR-3 in Thy-1/CD90-induced adhesion, polarization, and migration was explored.

**Fig. 2:**
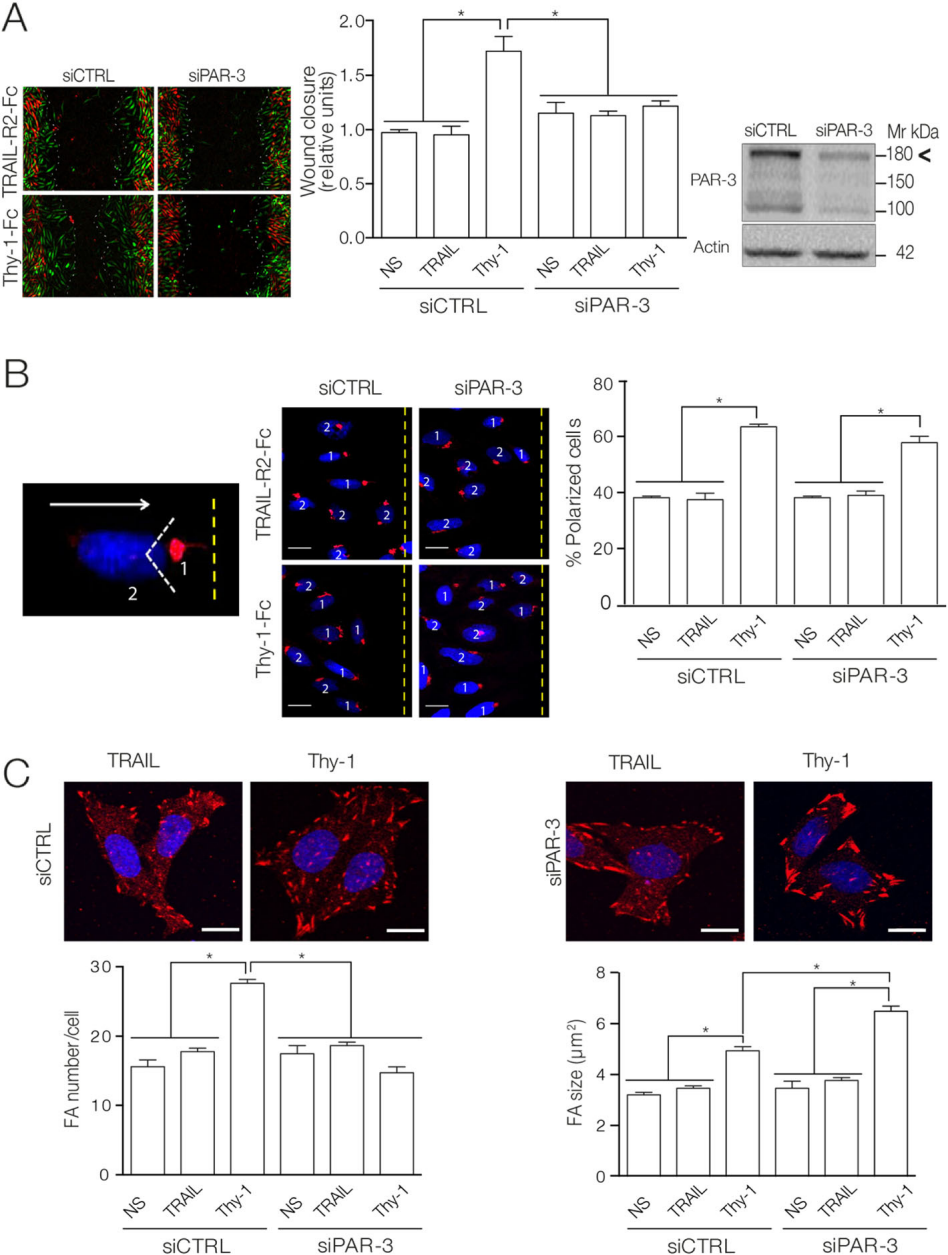
PAR-3 knockdown impaired astrocyte migration and adhesion, but not Golgi polarization induced by Thy-1. **a** Immunoblotting for PAR-3 isoforms in cells transfected with PAR-3 siRNA compared to control siRNA. Actin was the loading control. Migration was evaluated using the wound-healing assay. Evaluated were DI TNC1 cells with silenced PAR-3 or control, stimulated or not (NS) with Thy-1-Fc-Protein A (Thy-1) or TRAIL-R2-Fc-Protein A (TRAIL). Pictures show overlapping images of wound closure at time zero (red) and after 24 h (green). Dotted line shows the wound-closure boundary. Graph shows wound closure in relative units. **b** DI TNC1 astrocytes, transfected as in (**a**), were stimulated with Thy-1-Fc-Protein A (Thy-1) or TRAIL-R2-Fc-Protein A (TRAIL) for 7 h. After fixation, cells were stained for nucleus (DAPI) and Golgi apparatus using an anti-giantin antibody. Golgi (red) positioning within an angle of 120° in front of the nucleus (blue), facing the wound, was considered polarized (1). Non-polarized cells are labelled as (2). Arrow shows the direction of migration. One hundred cells were monitored per condition. Graph shows the percentage of polarized cells. Scale bar = 10 μm. **c** DI TNC1 astrocytes, transfected as in (**a**), were stimulated for 15 min. FAs were visualized by immunofluorescence using an anti-vinculin antibody (red) and DAPI for nuclei (blue). Scale bar = 10 μm. Image analyses were performed with the Analyze Particles Tool in the Fiji image processing software [44]. Values in the graphs represent the mean ± s.e.m. from 3 independent experiments (*n* = 3) determined from at least 50 cells in each experimental condition per experiment. Significant differences are indicated as *P < 0.05

First, PAR-3 full length (180 kDa) as well as its isoforms (150 and 100 kDa) were silenced using a siRNA pool (Fig. 2a). Cell migration was subsequently monitored in a wound-healing assay after 24 h of treatment with Thy-1-Fc. Cells with silenced PAR-3 isoforms and stimulated with Thy-1-Fc evidenced inhibited wound closure compared with the respective siRNA controls (Fig. 2a).

Second, cell polarization was evaluated using the same technique as above, but wound-healing was monitored after 7 h of Thy-1-Fc treatment. Golgi/nuclei staining distinguished non-polarized from polarized cells (i.e., Golgi apparatus in front of the nucleus and facing the wound border within a 120° angle [45] (Fig. 2b, left panel). PAR-3 silencing did not abolish Thy-1-Fc-induced Golgi polarization (Fig. 2b). This polarization process was also unaffected by PAR-3 silencing in non-stimulated and TRAIL-R2-Fc control cells (Fig. 2b).

Finally, cell adhesion was evaluated with a FA assembly assay, where an anti-vinculin antibody was employed to detect FAs by immunofluorescence. Our results show that PAR-3-silenced astrocytes stimulated for 30 min with Thy-1-Fc had fewer total FAs, but, unexpectedly, individual structures were larger and longer than in control cells (Fig. 2c). In contrast, Thy-1-Fc-stimulated astrocytes in siRNA control cells presented more and larger FAs than did non-stimulated and TRAIL-R2-treated control cells (Fig. 2c).

These results show that PAR-3 participates in the signaling pathways required for adhesion and migration. However, in agreement with a previous report that used astrocytes stimulated with FBS [27], PAR-3 in our model system was not involved in Golgi polarization.

### PAR-3 regulates FA disassembly and favors FAK dephosphorylation

**Fig. 3:**
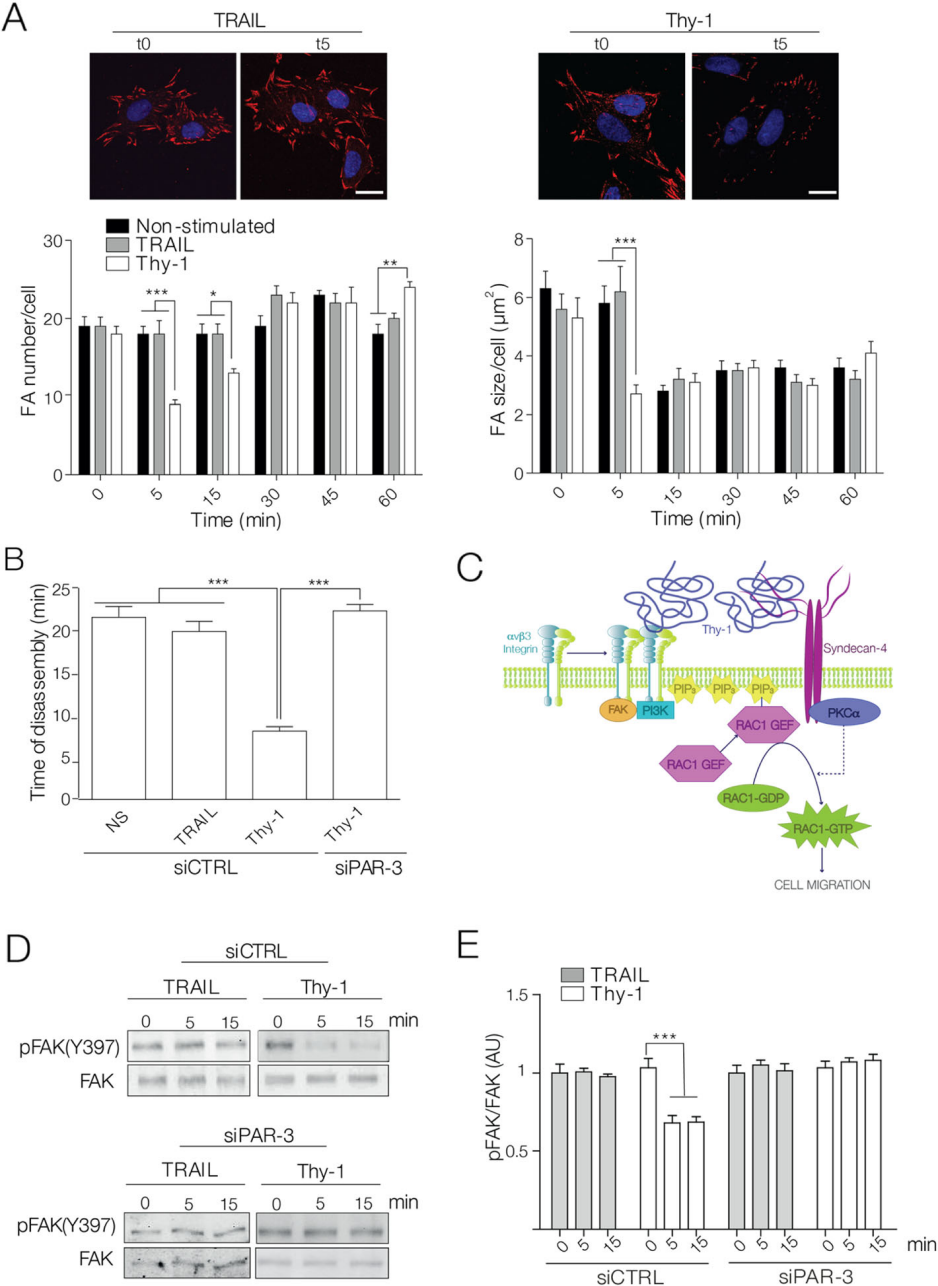
PAR-3 silencing impaired Thy-1-accelerated microtubule-dependent focal adhesion disassembly. **a** DI TNC1 cells treated with Nocodazole and washout were stimulated or not (NS) with Thy-1-Fc-Protein A (Thy-1) or TRAIL-R2-Fc-Protein A (TRAIL) for different time periods. FAs were detected by immunofluorescence staining for vinculin (red) and nuclei (blue). Scale bar = 10 μm. Graphs show quantification of the FA number per cell (left panel) and average area of FAs (right panel) observed in DI TNC1 astrocytes after the indicated treatments. Values in the graphs represent the mean ± s.e.m. determined from at least 50 cells in each experimental condition per experiment. **b** DI TNC1 astrocytes co-transfected with mCherry-vinculin and siRNA (control or PAR-3) were treated with Nocodazole and washout. Cells were then stimulated as in (**a**), and a 30-min time-lapse microscopy video was recorded. Values in the graphs represent the mean ± s.e.m. determined from scoring at least 10 mCherry-positive focal adhesions per experiment. **c** Model depicting the interaction of αvβ3 integrin and Syndecan-4 with Thy-1, which triggers signaling events that include activation of FAK and PI3K generating PIP_3_. An unidentified Rac1 GEF, is then recruited to the plasma membrane, where it activates Rac1 and leads to cell migration. **d** DI TNC1 astrocytes, transfected as in (**a**), were treated with Nocodazole and washout, and cells were stimulated for different time periods (0–15 min). Cell lysates were immunoblotted for p-FAK or total FAK. **e** Graph values are compared against the normalized time 0 and represent the average numbers obtained from the ratio of p-FAK/FAK densitometric data analysis. All graphs show the mean ± s.e.m. of *n* = 3. Significant differences compared to control conditions are indicated as *P < 0.05, **P < 0.01, and ***P < 0.001

Considering the surprising results indicating that larger and longer FAs were detected in PAR-3-knockdown cells, a possible role of PAR-3 in FA disassembly was evaluated. Cells were first treated with Nocodazole, a microtubule-depolymerizing drug [52] that blocks FA turnover [53, 54]*.* Nocodazole was washed out and cells were immediately stimulated with Thy-1-Fc; FA disassembly was then analyzed at different time points. Through vinculin staining, Thy-1-Fc-stimulated astrocytes evidenced more rapid FA disassembly (i.e., fewer and smaller FAs 5 min post-Nocodazole washout; Fig. 3a) than non-stimulated and TRAIL-R2-treated controls (i.e., no change in FA quantity, smaller FAs 15 min post-Nocodazole washout; Fig. 3a).

Since Thy-1-Fc speeds up FA disassembly after Nocodazole wash-out, we wanted to analyze the role of PAR-3 in this process. For this, PAR-3-silenced astrocytes were treated with Nocodazole and analyzed using time-lapse video microscopy immediately after washout. FA disassembly was delayed (i.e., 22 ± 1 min) in Thy-1-Fc-stimulated, PAR-3-silenced cells as compared with Thy-1-Fc-stimulated, siRNA-control cells (i.e., 8 ± 1 min) (Fig. 3b) (Movie S1 and S2). For PAR-3-silenced cells that were non-stimulated or TRAIL-R2-treated, FA disassembly was undetectable during the 30-min recording (Movie S3 and S4). In turn, siRNA control cells that were non-stimulated or TRAIL-R2-treated showed FA disassembly within 20 ± 1 min (Fig. 3b). Taken together, the evidence indicates that PAR-3 regulates FA disassembly.

After identifying these key components in FA disassembly, investigation was conducted on a possible underlying signal transduction mechanism. Our previous reports indicate that αvβ3 integrin and Syndecan-4 bind to Thy-1/CD90 and trigger signaling events that include activation of FAK, and PI3K downstream of integrin activation [17]. Consequently, PIP_3_ increases at the plasma membrane allowing translocation of a Rac1 GEF that leads to the activation of Rac1 and cell migration, events that also involve engagement of Syndecan-4 and the activation of PKCα (Fig. 3c) [8, 17]*.*

Additional reports also indicate a role for FAK in FA turnover, where Y397-FAK autophosphorylation is an important molecular marker for events linked to the control of FA dynamics. While higher phosphorylation levels are observed when FAs are stabilized, Y397 dephosphorylation is associated with FA disassembly [55]*.* Here, we showed that FAK co-precipitates with Syndecan-4-HA (Fig. 1c). Therefore, the possible effects of Thy-1-Fc on FAK phosphorylation during FA disassembly were assessed by monitoring Y397-FAK autophosphorylation after Nocodazole washout at different time points. Aligning with accelerated FA disassembly promoted by Thy-1-Fc (Fig. 3b), FAK dephosphorylation was detected as early as 5 min after Nocodazole washout (Fig. 3d, top panels). However, in PAR-3-silenced cells, Thy-1-Fc stimulation showed no differences in Y397-FAK autophosphorylation levels (Fig. 3d, bottom panels). Data shown in Fig. 3d is quantified in Fig. 3e.

Therefore, these results indicate that PAR-3 controls FA disassembly and that the presence of PAR-3, during this process, favors Y397-FAK dephosphorylation.

### PAR-3 regulates Tiam1 activation during Thy-1-induced migration

**Fig. 4:**
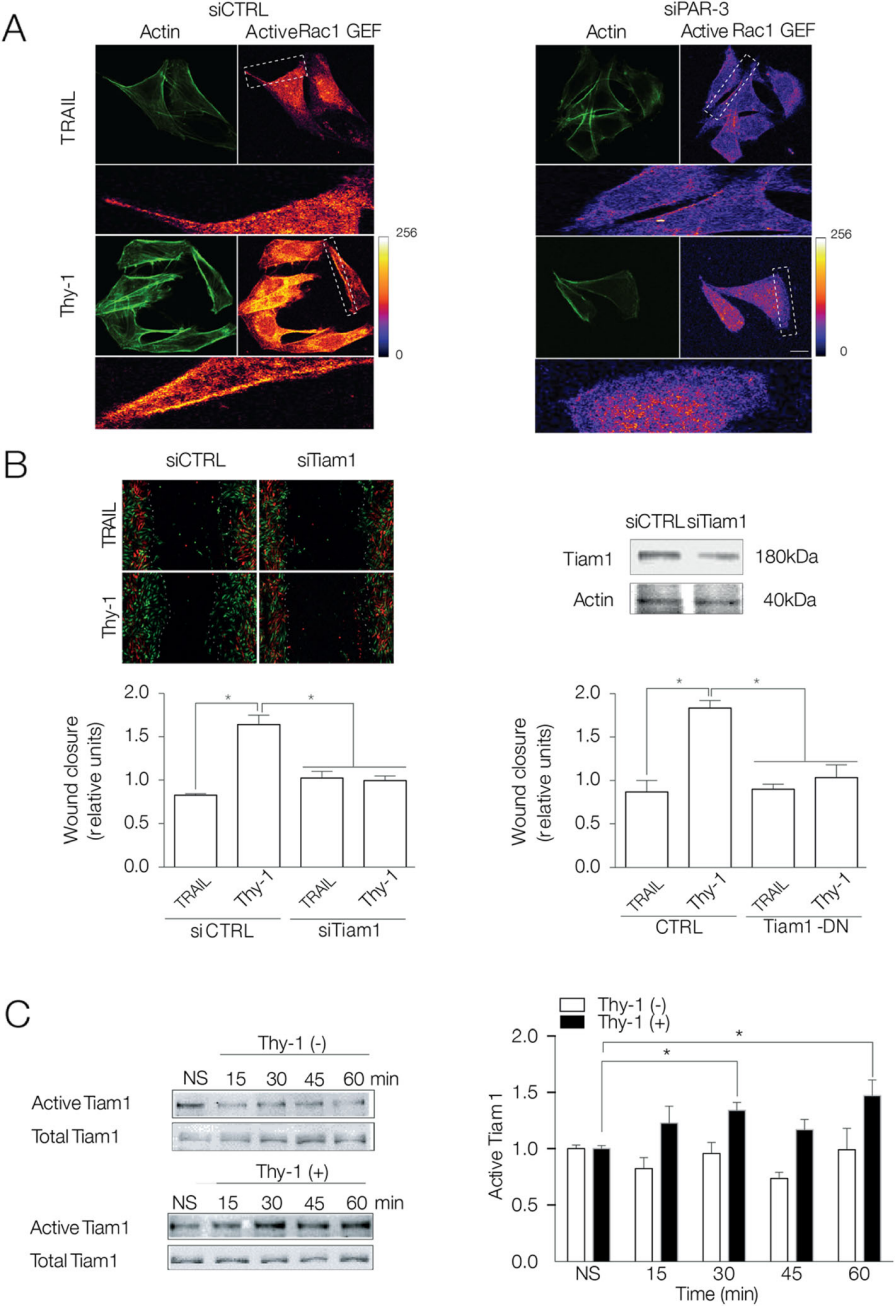
Thy-1-induced astrocyte migration requires PAR-3-dependent Tiam1 activation. **a** PAR-3 silenced DI TNC1 astrocytes were stimulated with Thy-1-Fc-Protein A (Thy-1) or TRAIL-R2-Fc-Protein A (TRAIL) for 60 min. Cells were fixed, and active Rac1 GEF was detected by immnunofluorescence using GST-Rac1G15A and anti-GST (purple) and Phalloidin (green). The white rectangle indicates the zoomed area. The color map indicates levels of Rac1 GEF activation, going from 0 (blue) to 256 (white). Scale bar = 10 μm. **b** DI TNC1 cells transfected with siRNA control (siCTRL), siRNA Tiam1 (siTiam1), or dominant negative Tiam1 (Tiam1-DN). The Western blot shows levels of Tiam1 in siRNA-transfected cells. Migration was evaluated using the wound-healing assay, and cells were stimulated as in (**a**) for 24 h. Images show wound closure at time zero (green) and after 24 h (red). **c** DI TNC1 cells stimulated or not (NS) with endogenous Thy-1 from fixed CAD cells for different periods of time. Active Tiam1 was affinity-precipitated using GST-Rac1G15A coupled to GSH-agarose beads and immunoblotted for Tiam1. Numbers in the graphs represent the average fold-increase of Tiam1 activity normalized to total Tiam-1. All graphs show the mean ± s.e.m. of *n* = 3. Significant differences compared to control conditions are indicated as *P < 0.05

Our previous work showed that Thy-1/CD90 engagement of αvβ3 integrin and Syndecan-4 activates Rac1 in astrocyte migration via an unknown Rac1 GEF (Fig. 3c) [17]. To further understand how PAR-3 is involved during Thy-1-induced migration, we first evaluated if PAR-3 participates in the activation of Rac1 GEFs using a modified Far-indirect immunofluorescence method [46]. This method was modified by using a nucleotide-free recombinant Rac1 protein (GST-Rac1G15A), which strongly binds to active Rac1 GEFs [47]. Therefore, GST-Rac1G15A was added to interact with active Rac1 GEFs present in cells expressing endogenous or reduced PAR-3 levels. As a result, higher levels of active Rac1 GEFs were detected in Thy-1-Fc-stimulated, siRNA-control cells than in TRAIL-R2-Fc cells (Fig. 4a, left panels). In contrast, cells with reduced PAR-3 levels had very low levels of activated Rac1 GEFs despite Thy-1-Fc or TRAIL-R2-Fc treatment (Fig. 4a, right panels).

Since PAR-3 can bind the GEF Tiam1 to activate Rac1 [56], we hypothesized that most of the active Rac1 GEFs observed in the Fig. 4a after Thy-1 stimulation could be Tiam1. To test this hypothesis, astrocytes were transfected with Tiam1-specific siRNA or with a dominant negative form of Tiam1 and wound-healing assays were conducted. Tiam1 silencing or dominant-negative expression prevented the enhancing effect of Thy-1-Fc on cell migration, suggesting that Tiam1 is required for Thy-1-Fc-induced astrocyte migration (Fig. 4b). Next, Tiam1 activation was confirmed by an affinity precipitation assay [17, 48]. Due to the amount of stimulus required to perform this assay, we used fixed CAD cells as a source of Thy-1/CD90 to stimulate astrocytes. This mode of activation has been previously validated by our group [17]. We found that CAD cells expressing Thy-1 on their surface induced Tiam1 activation in astrocytes after 30–60 min of treatment (Fig. 4c), which is in line with Tiam1 involvement in signaling pathways that promote astrocyte migration [17].

**Fig. 5:**
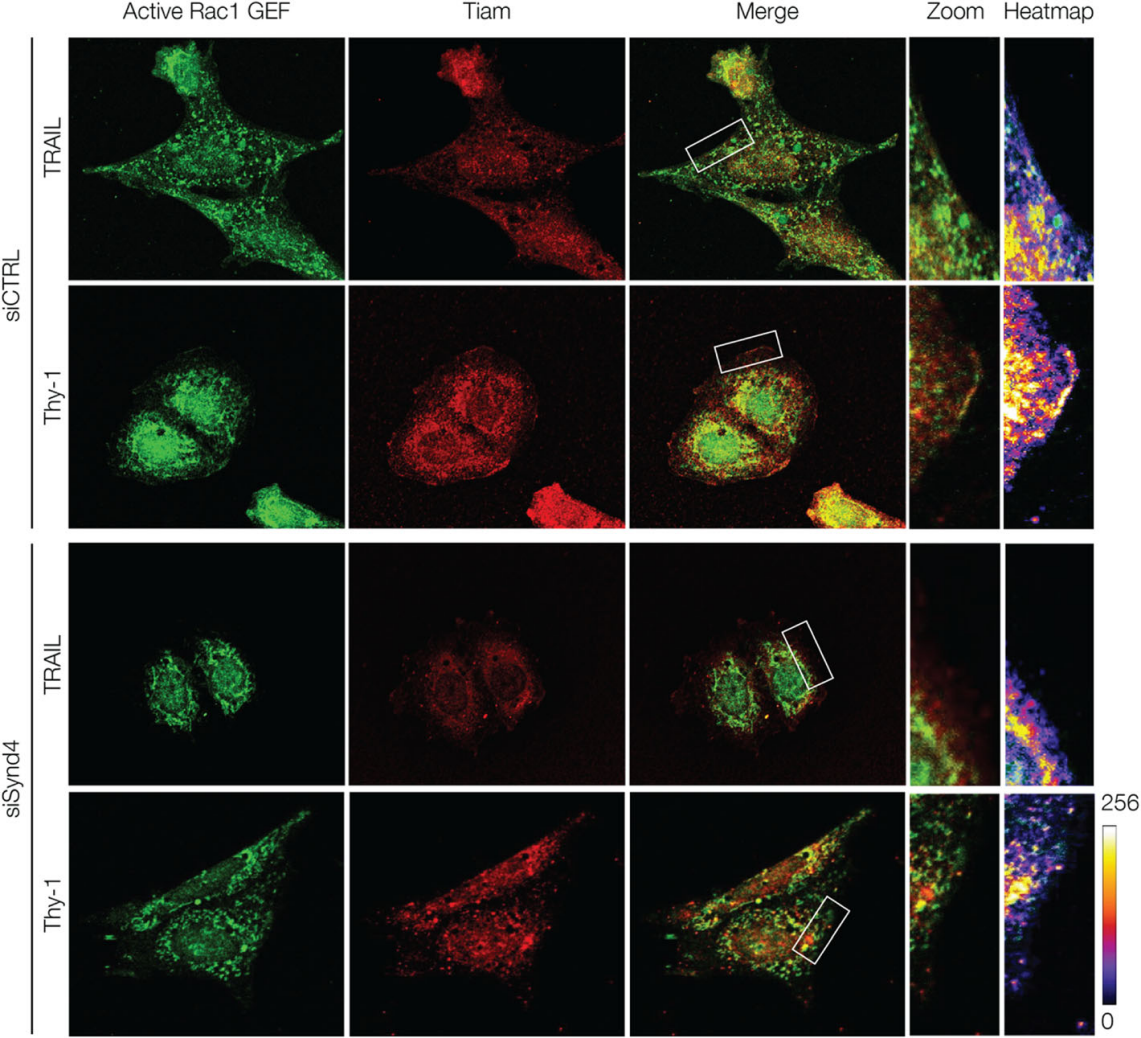
PAR-3-induced Tiam1 activation after Thy-1/CD90 treatment requires Syndecan-4. Syndecan-4silenced DI TNC1 astrocytes (siSynd4) or control transfected cells (siCTRL) were stimulated with Thy-1-Fc-Protein A (Thy-1) or treated with TRAIL-R2-Fc-Protein A (TRAIL) for 60 min. Cells were fixed, and stained for GST to detect GST-Rac1G15A (green) and for Tiam1 (red). The white rectangle indicates the zoomed area. The heat color map indicates levels of total Tiam1 co-localization with nucleotidefree mutant of Rac1 (GST-Rac1G15A) at the membrane, as indicative of Tiam1 activation. Scale bar = 10 μm

To prove that the active Rac1 GEF, identified in cells through GST-Rac1G15A binding, and Tiam1 coincide in cellular localization, we performed the same Far-indirect immunofluorescence experiment but, evaluated co-localization of anti-GST and anti-Tiam1 antibodies. More Tiam1 signal (red fluorescence) was detected at the plasma membrane of Thy-1-stimulated control cells (siCTRL) compared with TRAIL-R2-treated cells (Fig. 5, top panels, ±Thy-1-Fc), indicative of increased amount of active Tiam1 in cells stimulated with Thy-1/CD90. Importantly, the active Tiam1 signal coincided with the staining of the GST-Rac1G15A probe, suggesting that the active Rac1 GEF observed in Fig. 4a corresponds to Tiam1. Finally, we evaluated if Syndecan-4 was necessary for Tiam1 activation. To accomplish this, the same experiment was repeated in Syndecan-4-silenced cells. The co-localization between GST-Rac1G15A probe and Tiam1 observed in siCTRL cells does not occur when cells are depleted of Syndecan-4 (Fig. 5, bottom panels). Syndecan-4 silencing decreased cell spreading but did not affect cell viability (data not shown) in these experiments. These results agree with previous reports [8, 57, 58]. Therefore, these results suggest that active Tiam localizes to the plasma membrane in lamellipodia-like protrusions in a Thy-1/CD90- and Syndecan-4-dependent manner (Fig. 5, Zoom and heatmap boxes).

Altogether these results indicate that PAR-3 regulates cell migration by (i) associating with the adhesome protein Syndecan-4, (ii) favoring FAK dephosphorylation during FA disassembly, and (iii) regulating Tiam1 activation. Considering our previous report indicating the requirement of Rac1 activation for astrocyte migration [17], we suggest that Tiam1 is the missing Rac1 GEF that activates Rac1, downstream of integrin/Syndecan-4 engagement by Thy-1-Fc.

### Syndecan-4/PAR-3 signaling participates in FA disassembly in fibroblasts

**Fig. 6:**
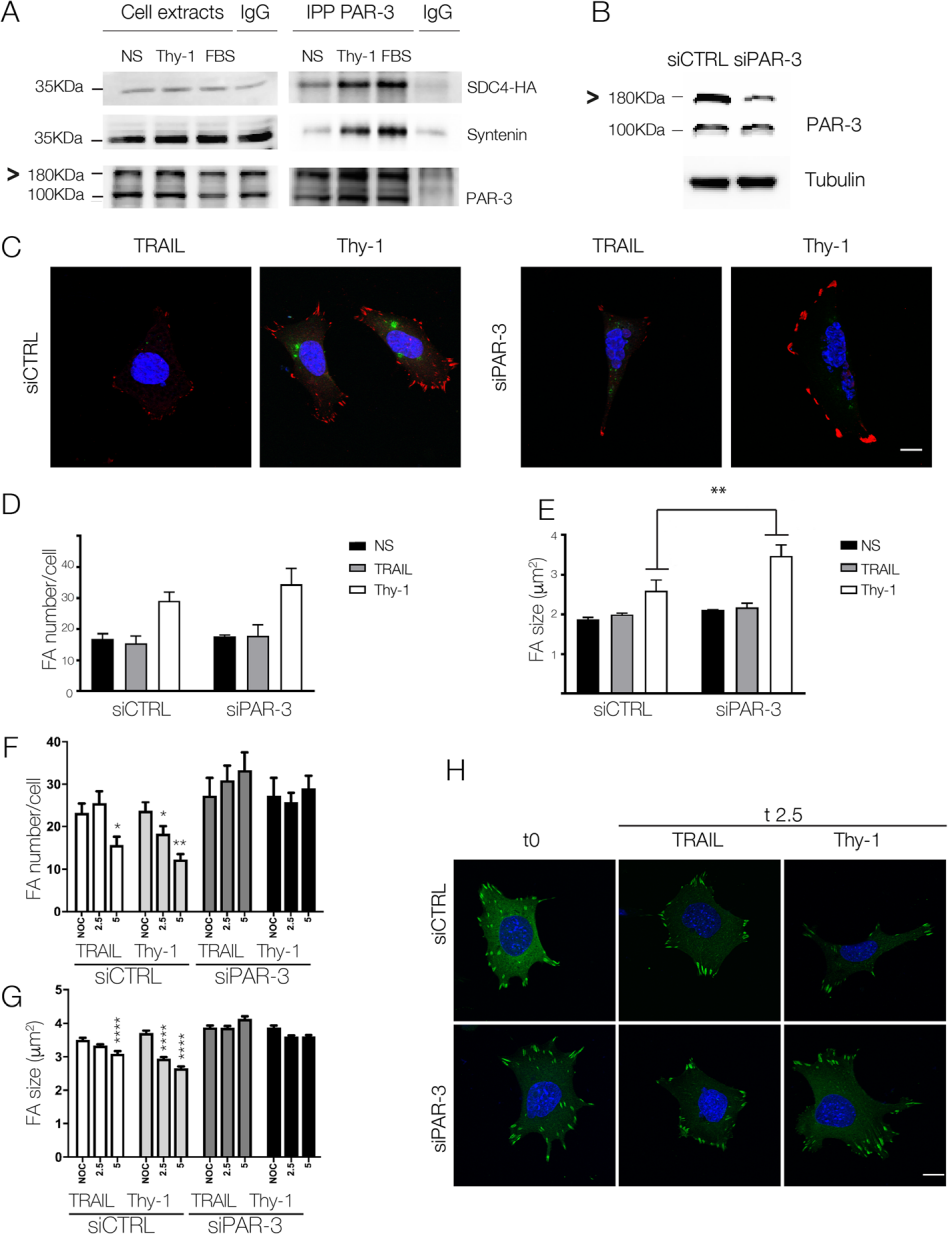
Thy-1-induced Syndecan-4/PAR-3 association is needed in FA dynamics in mouse embryonic fibroblasts. **a** MEFs transfected with Syndecan-4-HA were stimulated or not (NS) with Thy-1-Fc or fetal bovine serum (FBS). PAR-3 was immunoprecipitated (IP) from the lysates, and Syndecan-4 was detected with an anti-HA antibody (SDC4-HA). Syntenin and PAR-3 were immunoblotted as positive and an IP control, respectively. IgG was used as a negative IP control. **b** MEFs transfected with the siRNA control or PAR-3 were analyzed by Western blotting for PAR-3 expression. Tubulin was used as a loading control. **c** MEFs co-transfected with siGlo and siRNA control or PAR-3 were stimulated with Thy-1-Fc-Protein A (Thy-1) or TRAIL-R2-Fc-Protein A (TRAIL) for 15 min. FAs and nuclei were respectively visualized by vinculin staining (red) and DAPI (blue). Transfected cells were identified by the siGlo signal (green). Graphs show the mean ± s.e.m. for the number (**d**) and average area (**e**) of FAs from at least 50 cells. MEFs were transfected as in (**c**), incubated with Nocodazole and washout, and immediately stimulated as in (**c**) for different time periods. Graphs show the mean ± s.e.m. for the number (**f**) and average area (**g**) of FAs from at least 50 cells. Significant differences are indicated as *P < 0.05, **P < 0.01, and ***P < 0.001. (**h**) Cells were co-transfected with Paxillin-EGFP and siRNA (control or PAR-3). Shown are representative images for non-stimulated cells (t0) and cells stimulated with Thy-1-Fc-Protein A (Thy-1) or TRAIL-R2-Fc-Protein A (TRAIL) for 2.5 min (t2.5). Scale bar =10 μm

Once the existence of the Syndecan-4/PAR-3 complex was established, specifically in relation to controlling cell migration in astrocytes, the presence and functionality of this complex was tested in a different mesenchymal cell, namely mouse embryonic fibroblasts (MEFs). Unlike the employed astrocyte cell line, MEFs are primary cells immortalized and used for few passages only; therefore, they are a complementary mesenchymal cell model. PAR-3 was immunoprecipitated in MEFs expressing Syndecan-4-HA, and Syndecan-4 co-immunoprecipitation was detected by immunoblotting. Syndecan-4 interacted with PAR-3 in non-stimulated cells, as well as in Thy-1-Fc or FBS-stimulated cells (Fig. 6a). Of note, the amount of Syndecan-4 precipitated with PAR-3 was higher in Thy-1-Fc or FBS-stimulated cells (Fig. 6a) compared with non-stimulated cells. Similarly, Syntenin recruitment to immunoprecipitated Syndecan-4 was also induced by stimulation (Fig. 6a). These findings indicate that the Syndecan-4/PAR-3 complex exists in MEFs and that the amount of PAR-3 and Syndecan-4 complex formation increases after either Thy-1-Fc or FBS stimulation.

As previously shown for astrocytes, the total quantity and average individual area of FAs increased in control cells after Thy-1-Fc stimulation and in the absence of PAR-3 these FAs were larger (Fig. 2c). In fibroblasts, PAR-3 silencing (Fig. 6b) resulted in cells with a similar number of FAs after Thy-1-Fc stimulation (Fig. 6c and d), but with significantly larger FAs in Thy-1-Fc-stimulated (3.47 μm^2^; Fig. 6e) than control MEFs (2.6 μm^2^; Fig. 6e).

To further corroborate the effect of PAR-3 in MEFs during FA disassembly, MEFs with silenced PAR-3 were treated with Thy-1-Fc or TRAIL-R2-Fc immediately after Nocodazole washout, and FAs were analyzed. FA disassembly was accelerated in control cells stimulated with Thy-1-Fc (beginning at 2.5 min, peak at 5 min; Fig. 6f) compared to control cells treated with TRAIL-R2-Fc (beginning at 5 min; Fig. 6f). At 2.5 min in these control cells, FAs were less abundant and smaller in size in Thy-1-Fc-stimulated than in TRAIL-R2-Fc-treated MEFs (Fig. 6f, g and h). In line with the results obtained in astrocytes, this effect was not observed after silencing PAR-3, a condition in which FAs did not disassemble in cells treated either with Thy-1-Fc or TRAIL-R2-Fc, at least during the observation period (Fig. 6f, g and h).

Therefore, these results confirm that PAR-3 is needed for Thy-1-Fc-induced FA disassembly in MEFs. In addition to the aforementioned findings, this outcome demonstrates that Syndecan-4/PAR-3 binding, as part of the adhesome complex, is a common mechanism used by mesenchymal cells, not just astrocytes, to promote polarized cell migration.

## Discussion

Fully discovering the signaling pathways that govern cell migration is essential for understanding important processes, such as scarring and wound-healing. Syndecan proteins and syndecan signaling partners play key roles in these processes. Nevertheless, the contributions of syndecan signaling partners to cell adhesion and migration are poorly characterized. This study is the first to report on the participation of Syndecan-4/PAR-3 signaling in FA disassembly in mesenchymal cells, specifically astrocytes and fibroblasts, two cell-types that play a key role in scar formation [31, 32].

One novel research finding was that the adapter protein PAR-3 is an associated member of the adhesome and a partner for Syndecan-4. The EFYA sequence in the C-terminal region of syndecan proteins binds with PDZ domain-containing proteins [23, 25, 26]. Several syndecan-binding proteins containing PDZ domains, such as Syntenin, CASK, Synectin, Synbindin, and Tiam1, have been previously described. In particular, PAR-3 contains three PDZ domains [59]. These domains allow PAR-3 to interact with several proteins, including cell-cortex proteins associated with the cytoskeleton [18, 60]. The present study is the first to directly investigate the interaction between Syndecan-4 and PAR-3; moreover, we showed that its interaction depends on the EFYA sequence present in the Syndecan-4 tail. The obtained findings sustain that a Syndecan-4/PAR-3 interaction exists, thus providing new insights into the signaling mechanisms downstream of Syndecan-4 and into the role of PAR-3 in polarized cell migration. A noticeable feature of Syndecan-4 is that it signals forming oligomers [10], thus Syndecan-4 could bind independently to PAR-3, Syntenin, Tiam1, and FAK bringing together all these proteins in a signaling complex.

All PAR-3 spliced variants (i.e., 180 kDa, 150 kDa, and 100 kDa) have the three PDZ domains, so they can potentially bind Syndecan-4 through its EFYA sequence. Our work was centered on the PAR-3 full length protein since this contains all signaling modules described to play a role during cell polarization. However, we cannot disregard that there was a differential precipitation of the 150 and 100 kDa isoforms while using Syndecan-4-HA compared with biotinylated peptides. Moreover, the expression of those isoforms seems to vary in total lysates between cell types. Further experiments are necessary to determine if there are other domains either in Syndecan-4 or PAR-3 sequences that modulate the binding through the terminal EFYA sequence or if the signaling is affected when different PAR-3 isoforms bind Syndecan-4, or even if cell confluency regulates the expression and/or stability of PAR-3 splicing variants.

We also observed that Syndecan-4/PAR-3 binding tends to increase after FBS or Thy-1 stimulation during the precipitation assays, but this increase was significantly bigger in PLA assays, suggesting that the interaction between Syndecan-4/PAR-3 may be lost after the rough precipitation conditions compared with milder conditions during in situ assays.

Another important finding was that PAR-3 is involved in cell adhesion and migration, but not polarization of astrocytes. Dissecting these biological processes is relevant for two primary reasons. First, PAR-3 has a recognized role in cell polarization as part of the PAR complex formed by PAR-6/aPKC. This complex generates and maintains epithelial and neuronal cell polarity [25], in addition to regulating polarized cell migration in several species and cell lines [23, 56, 61, 62]. However, the polarity complex of migrating astrocytes does not contain PAR-3 [27]. The latter results agree with ours in that PAR-3 silencing did not affect Golgi polarization. Second, Syndecan-4 localizes and regulates FA dynamics in cell adhesion and migration [36], and PAR-3 partially localizes to FAs, binding and activating FAK and PI3K to regulate adhesion and directional cell migration [21, 51]. Notwithstanding, the exact role of PAR-3 in these events was previously unclear.

Adding to the prior results, this study also found that PAR-3 is required for FAK dephosphorylation occurring concomitant to FA disassembly. Itoh et al. reported that the PDZ domains of PAR-3 interact with the FERM domain of FAK regulating adhesion-induced FAK phosphorylation in HeLa cells [51]. On the other hand, FAK autophosphorylation on the Y397 residue is required for FA dynamics and migration [55, 63]. These data consequently suggest that PAR-3 might play a key role in regulating the FAK phosphorylation turnover that occurs during FA assembly/disassembly. However, the spatiotemporal link of these events is unclear. We postulate that, as a result of Syndecan-4 association, PAR-3 is recruited to FAs to regulate FAK phosphorylation, and thus, FA turnover. This proposal is supported by the finding that i) FAK co-precipitates with PAR-3 and Syndecan-4, ii) the absence of PAR-3 leads to the formation of long and large FAs, and that iii) specifically, during FA disassembly the absence of PAR-3 prevents FAK dephosphorylation. The extensive list of PAR-3-binding partners includes phosphatases such as PTEN and SHP-2, both of which can subsequently dephosphorylate FAK [64–66]. However, the mechanism by which PAR-3 favors FAK dephosphorylation requires further investigation.

The current study further discovered that Syndecan-4/PAR-3 complex regulates Tiam1 activation in astrocytes. In the context of PAR-3, the small GTPase Rac1, can be regulated by the GEFs Tiam1 or TRIO [67]. Different research groups [56, 68] showed that PAR-3 interacts with Tiam1 to mediate Rac1 activation in migrating cells. In turn, PAR-3 sequesters TRIO in neural crest cell-cell contacts, thereby inhibiting TRIO-mediated Rac1 activation [67]. Related research data show that Thy-1/CD90 induces Rac1 activation to promote astrocyte migration [17], an effect that is inhibited by the Rac1 GEF inhibitor NSC23766. This inhibitor impairs the activity of both Tiam1 and TRIO [69]. A possible explanation is that Tiam1/TRIO might regulate Rac1 activity during astrocyte migration. The presently obtained data align with the proposed role for Tiam1 in astrocytes; however, none of the conducted experiments rule out a possible role for TRIO.

Another noteworthy finding of this study was that PAR-3 binds to Syndecan-4 and regulates FA disassembly in both astrocytes and embryonic fibroblasts. Minor differences were observed in FA dynamics between cell types that may be related to their own characteristics: MEFs basally produce less and smaller FA than astrocytes. Consequently, in MEFs lower Nocodazole concentrations are required to destabilize the MT network and FA disassembly occurs faster than in astrocytes. Similarly, since astrocytes FA are bigger, the effect of silencing PAR-3 on FA size may tend to make adhesions to overlap, making it difficult to detect differences in the number of FA. Moreover, PAR-3 localizes to different subcellular compartments, including the leading edge, FAs, and cell-cell contacts, as well as being more diffusely distributed in the cytoplasm [18, 51, 62]. PAR-3 localizes to cell-cell contacts in neural crest cells [67], epithelial cells [25, 64, 66, 67], and fibroblast monolayers [52, 62, 70]. In neural crest cells (another mesenchymal cell type), PAR-3 controls contact inhibition of locomotion [67], while in epithelial cells, PAR-3 is required to establish apico-basal cell polarity [25]. Importantly, the participation of PAR-3 in FA disassembly is a novel and previously unappreciated role of this protein in cellular behavior, one that could explain the activation of Rac1 at the leading edge of cells with polarized migration. In a wound-healing assay of a neural crest cell monolayer, contact inhibition of locomotion polarizes cells to the wounded area. Considering the results presented by Mayor and co-workers [67], it is plausible to speculate that the role of PAR-3 differs between cell-cell contact sites and the leading edge in neural crest cell and other mesenchymal-like cells, such as fibroblasts and astrocytes. PAR-3 could bind the Rac1 GEF (i.e. TRIO) in cell-cell contacts, thereby inhibiting Rac1 through Rac1 GEF sequestration and, ultimately, polarizing the cells to the wound. In contrast, PAR-3 at the leading edge would bind to the Rac1 GEF (i.e. Tiam1) to activate Rac1 and promote membrane protrusions and polarized cell migration. Different associated partners in each location could account for varied functions, or the distinct PAR-3 isoforms could regulate each function. Analysis of these possibilities requires further studies.

Considering that Syndecan-4 regulates FAK autophosphorylation, the activation of RhoA and Rac1 GTPases, lamellipodia formation, and cell adhesion and migration [8, 17, 43]; additionally that, PAR-3 binds Tiam1 and FAK [51, 56, 67, 68], together with the present findings that Syndecan-4 binds PAR-3 and PAR-3 participates in FA disassembly (which correlates with increased FAK dephosphorylation and Tiam1 activation), we propose an integrative, comprehensive model for the role of Syndecan-4/PAR-3 signaling in FA dynamics (Graphical Abstract). We postulate that the Syndecan-4/PAR-3 signaling complex recruits Tiam1 and FAK, possibly to promote FAK dephosphorylation and the localization and activation of Tiam1 near the plasma membrane. This model highlights the role of the Syndecan-4/PAR-3 complex and FAK in controlling FA dynamics. At the membrane, Tiam1 activates Rac1 and is implicated in cell migration [71], and whether it is also involved in FA dynamics by virtue of its association with the Syndecan-4/PAR-3 signaling complex or by binding to PIP_3_ at the plasma membrane (Fig. 3c) is yet to be investigated.

## Conclusions

In summary, our findings indicate that PAR-3 is a novel Syndecan-4-binding protein, and that the newly termed Syndecan-4/PAR-3 signaling complex participates in FA disassembly. Both observations are key findings for understanding the signaling pathways regulating the adhesion and migration of mesenchymal cells. This novel Syndecan-4/PAR-3 signaling pathway provides new insights into the signaling mechanisms downstream of Syndecan-4 and the role PAR-3 plays in polarized cell migration. Due to the importance of syndecans in cytoskeletal regulation, migration, and cell-cell as well as cell-matrix interactions [36, 72, 73], future studies addressing the novel Syndecan-4/PAR-3 pathway in processes such as wound-healing and scarring should be of major interest in efforts to treat fatal chronic wounds.

## Supplementary information

**Additional file 1.** Precipitation of Syndecan-4 binding Proteins for MS. (**A**) Syndecan-4 cytoplasmic tail contains three different regions named C1-V-C2. The peptides used to precipitate Syndecan-4 binding proteins contain the last 10 amino acids indicated (SDC4). In control peptides (CTRL) the last 4 amino acids, EFYA have been replaced to four glycine. (**B**) Protein extracts from DI TNC1 cells, stimulated or not (NS) with fetal bovine serum (FBS), were precipitated with either biotinylated peptides of the Syndecan-4 (SDC4)-C-terminal sequence, which includes the C2 sequence, or the control peptide (CTRL). Precipitates were size-fractionated by 4–12% SDS-PAGE and silver stained. Bands differentially precipitated with SDC4 peptides were cut from the gel and analyzed by MS as described in Material and Methods. Relative molecular weight (Mr) is indicated to the right in kDa. The symbols (*) and (*’) indicate the bands identified as PAR-3 and Syntenin, respectively.

**Additional file 2. Movie S1.** siCTRL+Thy-1-Fc. Time lapse video of Thy-1-Fc-induced FA disassembly in cells expressing PAR-3. siControl-transfected DI TNC1 cells were co-transfected with mCherry-vinculin for 48 h and then incubated with 10 μM Nocodazole for 4 h. The Nocodazole was removed, and the samples were recorded for 30 min during stimulation with Thy-1-Fc.

**Additional file 3. Movie S2.** siPAR-3+ Thy-1-Fc. Time lapse video of Thy-1-Fc-induced FA disassembly in cells with decreased PAR-3 levels. siPAR-3-transfected DI TNC1 cells were co-transfected with mCherry-vinculin for 48 h and then incubated with 10 μM Nocodazole for 4 h. The Nocodazole was removed, and the samples were recorded for 30 min during stimulation with Thy-1-Fc.

**Additional file 4. Movie S3.** siCTRL+TRAIL-R2-Fc. Time lapse video of control FA disassembly. siControl-transfected DI TNC1 cells were co-transfected with mCherry-vinculin for 48 h and then incubated with 10 μM Nocodazole for 4 h. The Nocodazole was removed, and the samples were recorded for 30 min during treatment with TRAIL-R2-Fc.

**Additional file 5. Movie S4.** siPAR-3+ TRAIL-R2-Fc. Time lapse video of control FA disassembly in cells with decreased PAR-3 levels. siPAR-3-transfected DI TNC1 cells were co-transfected with mCherry-vinculin for 48 h and then incubated with 10 μM Nocodazole for 4 h. The Nocodazole was removed, and the samples were recorded for 30 min during treatment with TRAIL-R2-Fc.

## Data Availability

The datasets used and/or analyzed during the current study are available from the corresponding author on reasonable request. All fusion proteins utilized in this study must be obtained through Material Transfer Agreement.

## Abbreviations

Thy-1: Thymus cell antigen 1
CD90: Cluster of differentiation 90
TRAIL-R2: Tumor Necrosis Factor-Related Apoptosis Inducing Ligand -Receptor 2
FA: Focal Adhesion
PAR-3: Partitioning-defective 3
aPKC: atypical Protein Kinase C
GEFs: Guanine nucleotide exchange factors
PDZ: Post synaptic density protein, *Drosophila* disc large tumor suppressor, and zonula occludens-1 protein
FAK: Focal Adhesion Kinase

## References

[1] Dubash AD, Menold MM, Samson T, Boulter E, García-Mata R, Doughman R, et al. Chapter 1 focal adhesions: new angles on an old structure. Int Rev Cell Mol Biol. 2009;1:1–65. 10.1016/S1937-6448(09)77001-7 19766966

[2] Zaidel-Bar R, Itzkovitz S, Ma’ayan A, Iyengar R, Geiger B (2007). Functional atlas of the integrin adhesome. Nat Cell Biol.

[3] Horton ER, Byron A, Askari JA, Ng DHJ, Millon-Frémillon A, Robertson J, et al. Definition of a consensus integrin adhesome and its dynamics during adhesion complex assembly and disassembly. Nat Cell Biol. 2015;17:1577–87. 10.1038/ncb3257 PMC4663675 26479319

[4] Ridley AJ (1999). Stress fibres take shape. Nat Cell Biol.

[5] Schwartz MA, Horwitz AR (2006). Integrating adhesion, protrusion, and contraction during cell migration. Cell.

[6] Leyton L, Schneider P, Labra CV, Rüegg C, Hetz CA, Quest AFG (2001). Thy-1 binds to integrin β3 on astrocytes and triggers formation of focal contact sites. Curr Biol.

[7] Herrera-Molina R, Frischknecht R, Maldonado H, Seidenbecher CI, Gundelfinger ED, Hetz C, et al. Astrocytic αVβ3 integrin inhibits neurite outgrowth and promotes retraction of neuronal processes by clustering Thy-1. Hassan BA, editor. PLoS One. 2012;7:e34295. 10.1371/journal.pone.0034295 PMC3316703 22479590

[8] Avalos AM, Valdivia AD, Munoz N, Herrera-Molina R, Tapia JC, Lavandero S, et al. Neuronal Thy-1 induces astrocyte adhesion by engaging syndecan-4 in a cooperative interaction with αVβ3 integrin that activates PKCα and RhoA. J Cell Sci. 2009;122:3462–71. 10.1242/jcs.034827 PMC2746130 19723805

[9] Woods A, Couchman JR (2001). Syndecan-4 and focal adhesion function. Curr Opin Cell Biol.

[10] Elfenbein A, Simons M (2013). Syndecan-4 signaling at a glance. J Cell Sci.

[11] Lee D, Oh ES, Woods A, Couchman JR, Lee W (1998). Solution structure of a syndecan-4 cytoplasmic domain and its interaction with phosphatidylinositol 4,5-bisphosphate. J Biol Chem.

[12] Oh S, Jang CG, Ho IK (1998). Activation of protein kinase C by phorbol dibutyrate potentiates [3H]MK-801 binding in rat brain slices. Brain Res.

[13] Bass MD, Roach KA, Morgan MR, Mostafavi-Pour Z, Schoen T, Muramatsu T (2007). Syndecan-4-dependent Rac1 regulation determines directional migration in response to the extracellular matrix. J Cell Biol.

[14] Bass MD, Williamson RC, Nunan RD, Humphries JD, Byron A, Morgan MR (2011). A Syndecan-4 hair trigger initiates wound healing through Caveolin- and RhoG-regulated integrin endocytosis. Dev Cell.

[15] Baciu PC, Goetinck PF. Protein kinase C regulates the recruitment of syndecan-4 into focal contacts. Mol Biol Cell. 1995;6:1503–13. 10.1091/mbc.6.11.1503 PMC301307 8589452

[16] Lim ST, Longley RL, Couchman JR, Woods A. Direct binding of syndecan-4 cytoplasmic domain to the catalytic domain of protein kinase Cα (PKCα) increases focal adhesion localization of PKCα. J Biol Chem. 2003;278:13795–802. 10.1074/jbc.M208300200 12571249

[17] Kong M, Muñoz N, Valdivia A, Alvarez A, Herrera-Molina R, Cárdenas A (1833). Thy-1-mediated cell-cell contact induces astrocyte migration through the engagement of αVβ3 integrin and syndecan-4. Biochim Biophys Acta, Mol Cell Res.

[18] Grootjans JJ, Zimmermann P, Reekmans G, Smets A, Degeest G, Dürr J, et al. Syntenin, a PDZ protein that binds syndecan cytoplasmic domains. Proc Natl Acad Sci U S A. 1997;94:13683–8. 10.1073/pnas.94.25.13683 PMC28366 9391086

[19] Couchman JR. Syndecans: proteoglycan regulators of cell-surface microdomains? Nat Rev Mol Cell Biol. 2003;4:926–37. 10.1038/nrm1257 14685171

[20] Ludford-Menting MJ, Oliaro J, Sacirbegovic F, Cheah ETY, Pedersen N, Thomas SJ, et al. A network of PDZ-containing proteins regulates T cell polarity and morphology during migration and immunological synapse formation. Immunity. 2005;22:737–48. 10.1016/j.immuni.2005.04.009 15963788

[21] Wang S, Watanabe T, Matsuzawa K, Katsumi A, Kakeno M, Matsui T, et al. Tiam1 interaction with the PAR complex promotes Talin-mediated Rac1 activation during polarized cell migration. J Cell Biol. 2012;199:331–45. 10.1083/jcb.201202041 PMC3471226 23071154

[22] Solecki DJ, Govek E-E, Hatten ME (2006). mPar6 alpha controls neuronal migration. J Neurosci.

[23] Goldstein B, Macara IG. The PAR proteins: fundamental players in animal cell polarization. Dev Cell. 2007;13:609–22. 10.1016/j.devcel.2007.10.007 PMC2964935 17981131

[24] Etienne-Manneville S (2008). Polarity proteins in glial cell functions. Curr Opin Neurobiol.

[25] Joberty G, Petersen C, Gao L, Macara IG (2000). The cell-polarity protein Par6 links Par3 and atypical protein kinase C to Cdc42. Nat Cell Biol.

[26] Etienne-Manneville S, Hall A. Cell polarity: Par6, aPKC and cytoskeletal crosstalk. Curr Opin Cell Biol [Internet]. 2003;15:67–72. 10.1016/s0955-0674(02)00005-4 12517706

[27] Etienne-Manneville S, Hall A (2001). Integrin-mediated activation of Cdc42 controls cell polarity in migrating astrocytes through PKCζ. Cell..

[28] Ubil E, Duan J, Pillai ICL, Rosa-Garrido M, Wu Y, Bargiacchi F (2014). Mesenchymal-endothelial transition contributes to cardiac neovascularization. Nature..

[29] Vedrenne N, Sarrazy V, Richard L, Bordeau N, Battu S, Billet F (2017). Isolation of astrocytes displaying Myofibroblast properties and present in multiple sclerosis lesions. Neurochem Res.

[30] Desmoulière A, Redard M, Darby I, Gabbiani G. Apoptosis mediates the decrease in cellularity during the transition between granulation tissue and scar. Am J Pathol. 1995;146:56–66. PMC1870783 7856739

[31] Desmoulière A, Darby IA, Gabbiani G. Normal and pathologic soft tissue remodeling: role of the myofibroblast, with special emphasis on liver and kidney fibrosis. Lab Investig. 2003;83:1689–707. 10.1097/01.lab.0000101911.53973.90 14691287

[32] Abd-El-Basset EM, Fedoroff S (1997). Upregulation of F-actin and alpha-actinin in reactive astrocytes. J Neurosci Res.

[33] Shechter R, Schwartz M. CNS sterile injury: just another wound healing? Trends Mol Med. 2013;19:135–43. 10.1016/j.molmed.2012.11.007 23279948

[34] Maldonado H, Calderon C, Burgos-Bravo F, Kobler O, Zuschratter W, Ramirez O, et al. Astrocyte-to-neuron communication through integrin-engaged Thy-1/CBP/Csk/Src complex triggers neurite retraction via the RhoA/ROCK pathway. Biochim Biophys Acta, Mol Cell Res. 2017;1864:243–54. 10.1016/j.bbamcr.2016.11.006 27842221

[35] Xu J. Preparation, culture, and immortalization of mouse embryonic fibroblasts. Curr Protoc Mol biol. 2005. Chapter 28. 10.1002/0471142727.mb2801s70 18265366

[36] Morgan MR, Hamidi H, Bass MD, Warwood S, Ballestrem C, Humphries MJ. Syndecan-4 phosphorylation is a control point for integrin recycling. Dev Cell. 2013;24:472–85. 10.1016/j.devcel.2013.01.027 PMC3605578 23453597

[37] Mendoza P, Ortiz R, Diaz J, Quest AFG, Leyton L, Stupack D, et al. Rab5 activation promotes focal adhesion disassembly, migration and invasiveness in tumor cells. J Cell Sci. 2013;126:3835–47. 10.1242/jcs.119727 PMC4074302 23813952

[38] Pasten C, Cerda J, Jausoro I, Court FA, Cáceres A, Marzolo MP. ApoER2 and Reelin are expressed in regenerating peripheral nerve and regulate Schwann cell migration by activating the Rac1 GEF protein, Tiam1. Mol Cell Neurosci. 2015;69:1–11. 10.1016/j.mcn.2015.09.004 26386179

[39] Hermosilla T, Muñoz D, Herrera-Molina R, Valdivia A, Muñoz N, Nham SU (2008). Direct Thy-1/αVβ3 integrin interaction mediates neuron to astrocyte communication. Biochim Biophys Acta, Mol Cell Res.

[40] Henriquez M, Herrera-Molina R, Valdivia A, Alvarez A, Kong M, Munoz N, et al. ATP release due to Thy-1-integrin binding induces P2X7-mediated calcium entry required for focal adhesion formation. J Cell Sci. 2011;124:1581–8. 10.1242/jcs.073171 PMC3078821 21502139

[41] Garcia-Mata R, Dubash AD, Sharek L, Carr HS, Frost JA, Burridge K. The nuclear RhoA exchange factor Net1 interacts with proteins of the Dlg family, affects their localization, and influences their tumor suppressor activity. Mol Cell Biol. 2007;27:8683–97. 10.1128/MCB.00157-07 PMC2169424 17938206

[42] Avalos AM, Arthur WT, Schneider P, Quest AFG, Burridge K, Leyton L. Aggregation of Integrins and RhoA activation are required for Thy-1-induced morphological changes in astrocytes. J Biol Chem. 2004;279:39139–45. 10.1074/jbc.M403439200 15220352

[43] Alvarez A, Lagos-Cabré R, Kong M, Cárdenas A, Burgos-Bravo F, Schneider P, et al. Integrin-mediated transactivation of P2X7R via hemichannel-dependent ATP release stimulates astrocyte migration. Biochim Biophys Acta, Mol Cell Res. 2016;1863:2175–88. 10.1016/j.bbamcr.2016.05.018 27235833

[44] Schindelin J, Arganda-Carreras I, Frise E, Kaynig V, Longair M, Pietzsch T (2012). Fiji: an open-source platform for biological-image analysis. Nat Methods.

[45] Urra H, Torres VA, Ortiz RJ, Lobos L, Díaz MI, Díaz N, et al. Caveolin-1-enhanced motility and focal adhesion turnover require Tyrosine-14 but not accumulation to the rear in metastatic cancer cells. Yang BB, editor. PLoS One. 2012;7:e33085. 10.1371/journal.pone.0033085 PMC3323582 22505999

[46] Bustos MA, Lucchesi O, Ruete MC, Mayorga LS, Tomes CN. Rab27 and Rab3 sequentially regulate human sperm dense-core granule exocytosis. Proc Natl Acad Sci U S A. 2012;109:E2057–66. 10.1073/pnas.1121173109 PMC3409754 22753498

[47] Arthur WT, Ellerbroek SM, Der CJ, Burridge K, Wennerberg K (2002). XPLN, a guanine nucleotide exchange factor for RhoA and RhoB, but not RhoC. J Biol Chem.

[48] García-Mata R, Wennerberg K, Arthur WT, Noren NK, Ellerbroek SM, Burridge K. Analysis of activated GAPs and GEFs in cell lysates. Methods Enzymol. 2006;1:425–37. 10.1016/S0076-6879(06)06031-9 16472675

[49] Choi Y, Yun J-H, Yoo J, Lee I, Kim H, Son H-N, et al. New structural insight of C-terminal region of Syntenin-1, enhancing the molecular dimerization and inhibitory function related on Syndecan-4 signaling. Sci Rep. 2016;6:36818. 10.1038/srep36818 PMC5103296 27830760

[50] Lagos-Cabré R, Alvarez A, Kong M, Burgos-Bravo F, Cárdenas A, Rojas-Mancilla E, et al. αVβ3 integrin regulates astrocyte reactivity. J Neuroinflammation. 2017;14:194. 10.1186/s12974-017-0968-5 PMC5622429 28962574

[51] Itoh N, Nakayama M, Nishimura T, Fujisue S, Nishioka T, Watanabe T (2010). Identification of focal adhesion kinase (FAK) and phosphatidylinositol 3-kinase (PI3-kinase) as Par3 partners by proteomic analysis. Cytoskeleton..

[52] Liao G, Nagasaki T, Gundersen GG (1995). Low concentrations of nocodazole interfere with fibroblast locomotion without significantly affecting microtubule level: implications for the role of dynamic microtubules in cell locomotion. J Cell Sci.

[53] Ezratty EJ, Partridge MA, Gundersen GG (2005). Microtubule-induced focal adhesion disassembly is mediated by dynamin and focal adhesion kinase. Nat Cell Biol.

[54] Ezratty EJ, Bertaux C, Marcantonio EE, Gundersen GG (2009). Clathrin mediates integrin endocytosis for focal adhesion disassembly in migrating cells. J Cell Biol.

[55] Hamadi A. Regulation of focal adhesion dynamics and disassembly by phosphorylation of FAK at tyrosine 397. J Cell Sci. 2005;118:4415–25. 10.1242/jcs.02565 16159962

[56] Nishimura T, Yamaguchi T, Kato K, Yoshizawa M, Nabeshima Y, Ohno S, et al. PAR-6–PAR-3 mediates Cdc42-induced Rac activation through the Rac GEFs STEF/Tiam1. Nat Cell Biol. 2005;7:270–7. 10.1038/ncb1227 15723051

[57] Chelariu-Raicu A, Wilke C, Brand M, Starzinski-Powitz A, Kiesel L, Schüring AN (2016). Syndecan-4 expression is upregulated in endometriosis and contributes to an invasive phenotype. Fertil Steril.

[58] Vuong TT, Reine TM, Sudworth A, Jenssen TG, Kolset SO. Syndecan-4 is a major Syndecan in primary human endothelial cells in vitro, modulated by inflammatory stimuli and involved in wound healing. J Histochem Cytochem. 2015;63:280–92. 10.1369/0022155415568995 PMC4374057 25575567

[59] Liu X, Shepherd TR, Murray AM, Xu Z, Fuentes EJ. The structure of the Tiam1 PDZ domain/ Phospho-Syndecan1 complex reveals a ligand conformation that modulates protein dynamics. Structure. 2013;21:342–54. 10.1016/j.str.2013.01.004 PMC4086710 23395182

[60] Lin D, Edwards AS, Fawcett JP, Mbamalu G, Scott JD, Pawson T (2000). A mammalian PAR-3 – PAR-6 complex implicated in Cdc42 / Rac1 and aPKC signalling and cell polarity. Nat Cell.

[61] Kemphues K. PARsing embryonic polarity. Cell. 2000;101:345–8. 10.1016/s0092-8674(00)80844-2 10830161

[62] Schmoranzer J, Fawcett JP, Segura M, Tan S, Vallee RB, Pawson T, et al. Par3 and dynein associate to regulate local microtubule dynamics and centrosome orientation during migration. Curr Biol. 2009;19:1065–74. 10.1016/j.cub.2009.05.065 PMC2749750 19540120

[63] Papusheva E, de Queiroz FM, Dalous J, Han Y, Esposito A, Jares-Erijmanxa EA (2009). Dynamic conformational changes in the FERM domain of FAK are involved in focal-adhesion behavior during cell spreading and motility. J Cell Sci.

[64] Wu H, Feng W, Chen J, Chan L-N, Huang S, Zhang M. PDZ domains of PAR-3 as potential phosphoinositide signaling integrators. Mol Cell. 2007;28:886–98. 10.1016/j.molcel.2007.10.028 18082612

[65] Feng W, Wu H, Chan L-N, Zhang M. PAR-3-mediated junctional localization of the lipid phosphatase PTEN is required for cell polarity establishment. J Biol Chem. 2008;283:23440–9. 10.1074/jbc.M802482200 18550519

[66] Zhang K, Zhao H, Ji Z, Zhang C, Zhou P, Wang L, et al. Shp2 promotes metastasis of prostate cancer by attenuating the PAR3/PAR6/aPKC polarity protein complex and enhancing epithelial-to-mesenchymal transition. Oncogene. 2016;35:1271–82. 10.1038/onc.2015.184 26050620

[67] Moore R, Theveneau E, Pozzi S, Alexandre P, Richardson J, Merks A (2013). Par3 controls neural crest migration by promoting microtubule catastrophe during contact inhibition of locomotion. Development..

[68] Pegtel DM, Ellenbroek SIJ, Mertens AEE, van der Kammen RA, de Rooij J, Collard JG. The PAR-Tiam1 complex controls persistent migration by stabilizing microtubule-dependent front-rear polarity. Curr Biol. 2007;17:1623–34. 10.1016/j.cub.2007.08.035 17825562

[69] Gao Y, Dickerson JB, Guo F, Zheng J, Zheng Y. Rational design and characterization of a Rac GTPase-specific small molecule inhibitor. Proc Natl Acad Sci. 2004;101:7618–23. 10.1073/pnas.0307512101 PMC419655 15128949

[70] Woods A, Couchman JR. Syndecan 4 heparan sulfate proteoglycan is a selectively enriched and widespread focal adhesion component. Mol Biol Cell. 1994;5:183–92. 10.1091/mbc.5.2.183 PMC301024 8019004

[71] Palamidessi A, Frittoli E, Garré M, Faretta M, Mione M, Testa I (2008). Endocytic trafficking of Rac is required for the spatial restriction of signaling in cell migration. Cell.

[72] Couchman JR, Woods A (1999). Syndecan-4 and integrins: combinatorial signaling in cell adhesion. J Cell Sci.

[73] Echtermeyer F, Baciu PC, Saoncella S, Ge Y, Goetinck PF (1999). Syndecan-4 core protein is sufficient for the assembly of focal adhesions and actin stress fibers. J Cell Sci.

