# Supplementary material for: Syndecan-4/PAR-3 signaling regulates focal adhesion dynamics in mesenchymal cells
Source: Cell Commun Signal. 2020 Aug 18;18:129. doi: 10.1186/s12964-020-00629-3 (PMC7433185; doi:10.1186/s12964-020-00629-3)
Supplement: Supplementary file 1 — Additional file 1. Precipitation of Syndecan-4 binding Proteins for MS. (A) Syndecan-4 cytoplasmic tail contains three different regions named C1-V-C2. The peptides used to precipitate Syndecan-4 binding proteins contain the last 10 amino acids indicated (SDC4). In control peptides (CTRL) the last 4 amino acids, EFYA have been replaced to four glycine. (B) Protein extracts from DI TNC1 cells, stimulated or not (NS) with fetal bovine serum (FBS), were precipitated with either biotinylated peptides of the Syndecan-4 (SDC4)-C-terminal sequence, which includes the C2 sequence, or the control peptide (CTRL). Precipitates were size-fractionated by 4–12% SDS-PAGE and silver stained. Bands differentially precipitated with SDC4 peptides were cut from the gel and analyzed by MS as described in Material and Methods. Relative molecular weight (Mr) is indicated to the right in kDa. The symbols (*) and (*’) indicate the bands identified as PAR-3 and Syntenin, respectively. [file 12964_2020_629_MOESM1_ESM.docx]

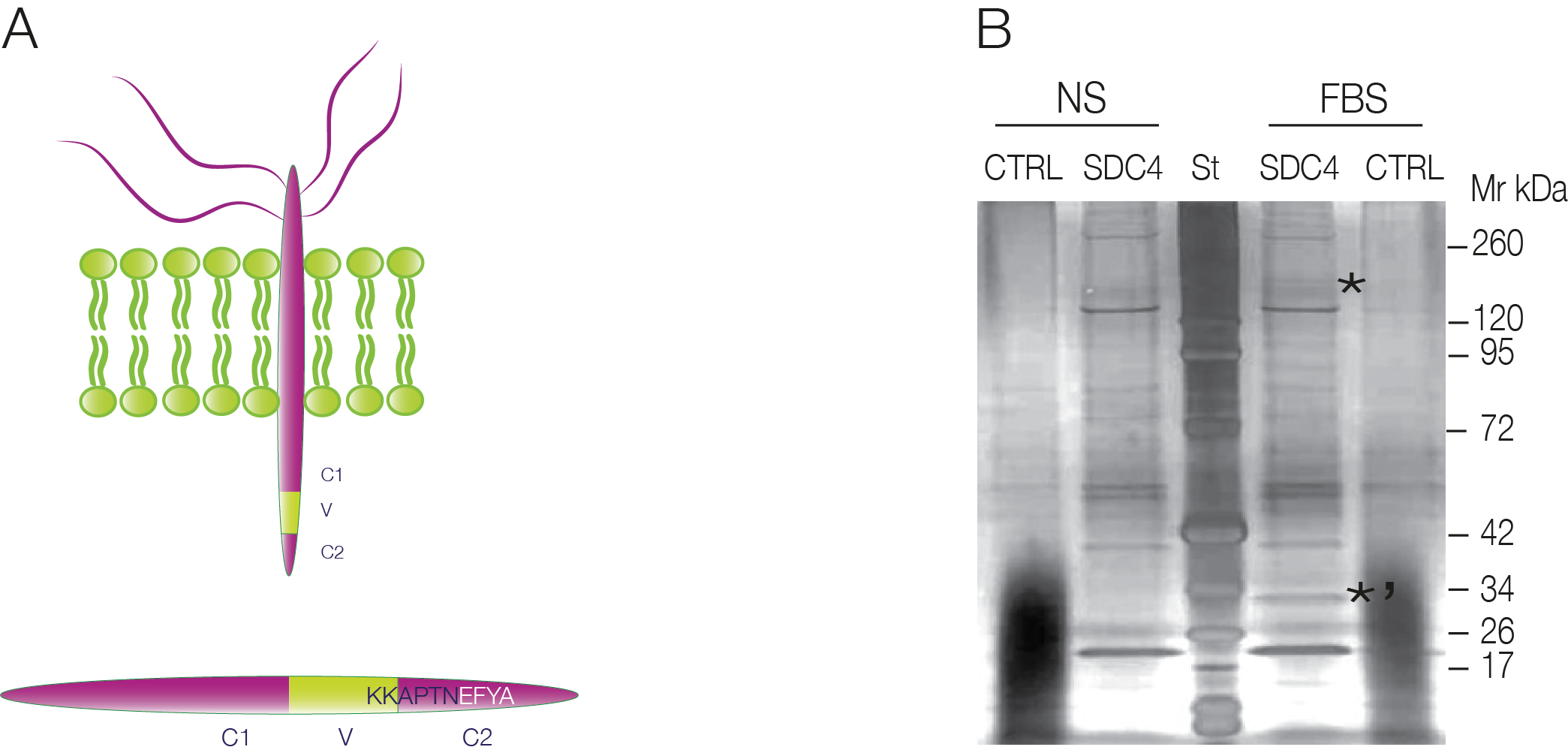


**Additional File 1**

**Precipitation of Syndecan-4 binding Proteins for MS.** (**A**) Syndecan-4 cytoplasmic tail contains three different regions named C1-V-C2. The peptides used to precipitate Syndecan-4 binding proteins contain the last 10 amino acids indicated (SDC4). In control peptides (CTRL) the last 4 amino acids, EFYA have been replaced to four glycine. (**B**) Protein extracts from DI TNC1 cells, stimulated or not (NS) with fetal bovine serum (FBS), were precipitated with either biotinylated peptides of the Syndecan-4 (SDC4)-C-terminal sequence, which includes the C2 sequence, or the control peptide (CTRL). Precipitates were size-fractionated by 4-12% SDS-PAGE and silver stained. Bands differentially precipitated with SDC4 peptides were cut from the gel and analyzed by MS as described in Material and Methods. Relative molecular weight (Mr) is indicated to the right in kDa. The symbols (*) and (*´) indicate the bands identified as PAR-3 and Syntenin, respectively.
